# Comparative genomics of the Leukocyte Receptor Complex in carnivores

**DOI:** 10.3389/fimmu.2023.1197687

**Published:** 2023-05-10

**Authors:** April L. Jelinek, Jan Futas, Pamela A. Burger, Petr Horin

**Affiliations:** ^1^ Department of Animal Genetics, Faculty of Veterinary Medicine, University of Veterinary Sciences Brno (VETUNI), Brno, Czechia; ^2^ Research Group Animal Immunogenomics, Central European Institute of Technology (CEITEC) VETUNI, Brno, Czechia; ^3^ Research Institute of Wildlife Ecology, University of Veterinary Medicine Vienna (VETMEDUNI), Vienna, Austria

**Keywords:** Leukocyte Receptor Complex, KIR, LILR, carnivora, long-read sequencing, felids

## Abstract

**Background:**

The mammalian Leukocyte Receptor Complex (LRC) chromosomal region may contain gene families for the killer cell immunoglobulin-like receptor (KIR) and/or leukocyte immunoglobulin-like receptor (LILR) collections as well as various framing genes. This complex region is well described in humans, mice, and some domestic animals. Although single KIR genes are known in some Carnivora, their complements of LILR genes remain largely unknown due to obstacles in the assembly of regions of high homology in short-read based genomes.

**Methods:**

As part of the analysis of felid immunogenomes, this study focuses on the search for LRC genes in reference genomes and the annotation of LILR genes in Felidae. Chromosome-level genomes based on single-molecule long-read sequencing were preferentially sought and compared to representatives of the Carnivora.

**Results:**

Seven putatively functional LILR genes were found across the Felidae and in the Californian sea lion, four to five genes in Canidae, and four to nine genes in Mustelidae. They form two lineages, as seen in the Bovidae. The ratio of functional genes for activating LILRs to inhibitory LILRs is slightly in favor of inhibitory genes in the Felidae and the Canidae; the reverse is seen in the Californian sea lion. This ratio is even in all of the Mustelidae except the Eurasian otter, which has a predominance of activating LILRs. Various numbers of LILR pseudogenes were identified.

**Conclusions:**

The structure of the LRC is rather conservative in felids and the other Carnivora studied. The LILR sub-region is conserved within the Felidae and has slight differences in the Canidae, but it has taken various evolutionary paths in the Mustelidae. Overall, the process of pseudogenization of LILR genes seems to be more frequent for activating receptors. Phylogenetic analysis found no direct orthologues across the Carnivora which corroborate the rapid evolution of LILRs seen in mammals.

## 1 Introduction

The mammalian immune system provides critical protection against a broad variety of insults but when dysregulated can itself give rise to pathologies. Its proper function and regulation depend critically on a balance of activating and inhibitory signals, which are received and coordinated *via* cell-surface immunoreceptors and their associated downstream signaling pathways. In vertebrates, the genes underlying these receptors are often found in clusters containing evolutionarily and/or functionally related genes and gene families (1), and these regions are frequently preserved across phylogeny (2). 

Although significant attention continues to be paid to the genetic mechanisms underlying the adaptive immune system, less attention has been given to the genetics of the innate immune system, which is essential to the survival of an organism and ultimately a species. Marked differences in the cell repertoire and the functioning of the innate immune system have been documented between closely related species (3, 4), and it has been shown that species within a family can vary in the degree to which they rely on the adaptive vs. innate immune systems to achieve immunocompetence (5). Among the cells with functions related to innate immunity, Natural Killer (NK) cells are notable for their capacity to lyse target host cells, an ability shared only by CD8+ T cells. This capability is significant not only for the elimination of intracellular pathogens but also for the destruction of cancer cells and can be harnessed for therapeutic purposes (6, 7).

Two genomic regions contain clusters of NK cell receptors (NKR) in mammals: the Natural Killer Complex (NKC) and the Leukocyte Receptor Complex (LRC). The NKR genes found in these complexes are structurally related within each complex but differ between the complexes. The NKC contains the killer-cell lectin-like receptors (KLRs) which possess C-type lectin-like extracellular domains, while the LRC genes encode receptors with extracellular ligand-binding domains belonging to the immunoglobulin (Ig) superfamily, such as the killer-cell immunoglobulin-like receptors (KIRs) and the leukocyte immunoglobulin-like receptors (LILRs) (1). Despite their structural differences, representatives of these gene families can bind polymorphic major histocompatibility complex (MHC) class I molecules as ligand. Moreover, they share similar transmembrane and intracellular domains, and therefore can fulfil the same functions in terms of downstream signaling and mediation of NK cell responses (8, 9). Specifically, these gene families code for both activating and inhibitory receptors, and ultimately the balance of activating and inhibitory signals received by a given NK cell determines its activation status and allows the cell to distinguish between self and non-self or altered self (10).

In contrast to the MHC, which has a relatively conserved genomic organization in mammals (11), the genomic regions containing the NKR gene families are evolutionarily flexible and rapidly evolving. In an interesting example of convergent evolution in mammals, different NKR gene families have expanded and diversified in different mammalian species (9, 12). NK cell MHC-I recognition is mediated by receptors encoded in the LRC in humans and higher primates (13), contrary to receptors encoded in the NKC in rodents (14), equids (15), and prosimians (16). The marked difference between mice and rats demonstrates that even within closely related species the gene content of the NKC and LRC can vary significantly.

Within the LRC, the KIR family has been characterized across a variety of placental mammals, with expansion and diversification of the KIR described in some species of primates (platyrrhines and catarrhines) and some artiodactyls (cattle) (9). The carnivores studied to date include domestic dogs, domestic cats, and several pinniped species (17). No expansion of the KIR gene family has been identified in any of these species: the domestic dog genome lacks KIR entirely, while a single KIR gene or pseudogene has been identified in domestic cats, three species of seals, and sea lions.

Less is known about the LILRs, which have mainly been studied in primates (18) and mice (as PIRs, the murine orthologues of LILRs) (19), as well as in several species of Cetartiodactyla, including pigs (20), goats (21), and cattle (22). Like other immunoreceptor gene families, the LILRs comprise both activating and inhibitory receptors (23). Activating LILRAs possess a short cytoplasmic tail and a positively charged amino acid residue in the transmembrane region which associates with immunoreceptor tyrosine-based activation motif (ITAM) containing proteins for signal transduction. Inhibitory LILRBs have long cytoplasmic tails containing 2 to 4 immunoreceptor tyrosine-based inhibitory motifs (ITIMs) (24). In addition, several LILRAB genes with characteristics typical of both activating and inhibitory LILRs have been described in primates (18), and one human LILR without transmembrane and signaling domains is known to be expressed as a secreted protein (25). Both LILRA and LILRB gene products comprise 2 to 6 extracellular Ig domains, depending on the species: either 2 or 4 Ig domains are typical for human LILRs (24), while 6 Ig domains are typical for murine PIRs (26) and can also be found in some bovine LILRs (22). Additionally, group 1 and group 2 LILRs have been defined for pigs, cattle, and goats based on phylogenetic analysis, with human LILRs clustering with the Cetartiodactyla group 2 LILRs (20).

LILRs are expressed in a variety of immune cells, including monocytes, macrophages, B and T lymphocytes, granulocytes, NK cells, mast cells, and dendritic cells (18). Although they have been shown to bind a variety of ligands, their interaction with MHC-I is the best described (27). Other ligands, especially for activating LILRs, include pathogen-associated proteins and host immunomodulatory molecules (28). However, at this time the function of various LILRs is poorly characterized, especially in non-human species. In humans and to a lesser extent in mice, LILRs (and their PIR orthologues) have been shown to play roles in autoimmune and inflammatory diseases, viral responses, neurodegenerative disorders, infectious diseases, and cancer (18, 29). Therefore, they are considered potentially useful as diagnostic markers and as a target for immunotherapies (30–33).

The family Felidae has the potential to be an informative model for comparative immunogenetic studies. Its members include species living in a variety of habitats and expressing a huge diversity of feeding and social behaviors, resulting in exposure to different pathogen pressures. Despite this potential, our knowledge of the immunogenomes of felids is limited, although more attention has been paid to domestic cats than wild felids. Nonetheless, differences in immune function between Felidae species have been documented, such as the increased reliance on innate immune mechanisms in cheetahs compared to a sympatric leopard population (5). Therefore, inferences from one species to another may be inaccurate, and the study of the immunogenome of individual species is warranted. A review of the MHC in Felidae was recently published, contributing to our knowledge of the genes underlying the adaptive immune system in these species (34). However, the genes underlying innate immune receptors, and particularly the LILRs, remain largely undescribed. The current study, therefore, aims to characterize the LRC and particularly the LILR gene family in the felid long-read, chromosome-level genome assemblies currently available.

## 2 Methods

### 2.1 Genomic resources

Ten long-read genomic assemblies at the chromosome level were available at NCBI (https://www.ncbi.nlm.nih.gov/genome) for felid species at the time of writing, and were the focus of the present study: cheetah (*Acinonyx jubatus*, GCA_027475565.2), domestic cat (*Felis catus*, GCA_018350175.1), jungle cat (*Felis chaus*, GCA_019924945.1), Geoffroy’s cat (*Leopardus geoffroyi*, GCA_018350155.1), Canada lynx (*Lynx canadensis*, GCA_007474595.2), clouded leopard (*Neofelis nebulosa*, GCA_028018385.1), lion (*Panthera leo*, GCA_018350215.1), tiger (*Panthera tigris*, GCA_018350195.2), Bengal cat (*Prionailurus bengalensis*, GCA_016509475.2), and fishing cat (*Prionailurus viverrinus*, GCA_022837055.1). These assemblies were all generated using single-molecule long-read (SMLR) technology from Pacific Biosciences. Assemblies that do not meet this criteria (short read or assembled only at the scaffold level) were available for 12 additional felid species: caracal (*Caracal caracal*, GCA_016801355.1), black-footed cat (*Felis nigripes*, GCA_004023925.1), Iberian lynx (*Lynx pardinus*, GCA_900661375.1), bobcat (*Lynx rufus*, GCA_022079265.1), Sunda clouded leopard (*Neofelis diardi*, GCA_027422475.1), manul (*Otocolobus manul*, GCA_028564725.1), jaguar (*Panthera onca*, GCA_004023805.1), leopard (*Panthera pardus pardus*, GCA_024362865.1), snow leopard (*Panthera uncia*, GCA_023721935.1), Iriomote cat (*Prionailurus iriomotensis*, GCA_018403415.1), puma (*Puma concolor*, GCA_003327715.1), and yagouaroundi (*Puma yagouaroundi*, GCA_014898765.1).

Short-read assemblies of additional Feliformia species for which it was possible to reconstruct the LRC across a series of scaffolds included the spotted hyena (*Crocuta crocuta*, GCA_008692635.1), the striped hyena (*Hyena hyena*, GCA_003009895.1), the aardwolf (*Proteles cristata cristata*, GCA_017311185.1), and the meercat (*Suricata suricatta*, GCA_06229205.1).

Available long-read chromosome-level assemblies for the Canidae were the domestic dog (*Canis lupus familiaris*, GCA_014441545.1), the dingo (*Canis lupus dingo*, GCA_003254725.2, combination of long- and short-read methodology), the gray wolf (*Canis lupus*, GCA_905319855.2), the Tibetan sand fox (*Vulpes ferrilata*, GCA_024500485.1), and the arctic fox (*Vulpes lagopus*, GCA_018345385.1). The Mustelidae were represented by the American mink (*Neogale vison*, GCA_020171115.1, combined methodology), the European badger (*Meles meles*, GCA_922984935.1), the Eurasian otter (*Lutra lutra*, GCA_902655055.2), and the Ermine (*Mustela erminea*, GCA_009829155.1, combined methodology). The final long-read chromosome-level assembly available for the Carnivora was that of the California sea lion (*Zalophus californianus*, GCA_009762305.2) as a sole representative of the Otariidae.

### 2.2 Bioinformatic tools

To clearly identify/distinguish LILR genes, non-LILR genes of the LRC were identified first and their putative functionality was assigned based on the NCBI´s Gnomon annotation (RNA-sequencing). In cases where such annotation was not available, the location and putative functionality of these genes was determined using Splign (https://www.ncbi.nlm.nih.gov/sutils/splign) with a “gold standard” mRNA model of that gene derived from the high quality and well annotated *Felis catus* reference assembly and checked to contain the appropriate conserved domains identified through comparison with their human orthologues. LILR genes were identified by the tBLASTn algorithm (https://blast.ncbi.nlm.nih.gov) using the four immunoglobulin (Ig) domains and the long cytoplasmic tail of the *F. catus LILRB6* (XP_023101497.1) as queries. The identified Ig domains and cytoplasmic tails were then manually described, based on their respective positions and proximity to one another in the assembly, as part of putative LILRs or other Ig domain-containing genes (e.g. *KIR*, *OSCAR*, *FCAR*, etc.) or as lone Ig domains not associated with any gene or pseudogene. BLAST, SignalP 6.0 (http://www.cbs.dtu.dk/services/SignalP/), and DeepTMHMM (https://dtu.biolib.com/DeepTMHMMM) were used to identify signal peptides (SP) and transmembrane domains (TM) associated with each gene. To be considered functional, a LILR gene was required to contain the following domains without frameshift or nonsense mutations: a SP, 2 or more Ig domains, a TM domain, and either a short cytoplasmic tail along with a positively charged amino acid (arginine) in the TM region (LILRA) or a long cytoplasmic tail containing one or more ITIMs (LILRB). Full-length LILR genes and pseudogenes were named according to the nomenclature system proposed by Schwartz and Hammond (20), with the caveat that clade numbers were omitted to simplify the names slightly. The alignment and putative functionality of the *LILR*, *KIR*, and *novel Ig-like* genes was checked by Splign using a gold standard mRNA model as described above. Again using Splign, coding sequences (CDS) were extracted for putative *LILR* genes and the *novel Ig-like gene* from genome assemblies of studied species to BioEdit version 7.2.6 (35).

The *F. catus* gold standard genes in the subregion containing the LILRs (ranging from *TTYH1* to *FCAR*, exclusive) served as the basis of comparison for felid LRCs using mVISTA (https://genome.lbl.gov/vista/index.shtml). In two cases, the yagouaroundi and the caracal, the LRC is split between several scaffolds and was reconstructed as a whole in BioEdit version 7.2.6 prior to being uploaded to VISTA. Exons and untranslated regions within these genes were identified in each felid assembly by Splign with the gold standard genes. The Shuffle-LAGAN alignment algorithm was used to identify and compensate for rearrangements within the analyzed sub-region.

The chromosomal rearrangement relative to other Mustelidae in *Neogale vison* and its potential impact on the LRC was explored by manual comparison of the annotated genes on each of the relevant chromosomes.

Phylogeny was sought based on CDS extracted from genome assemblies and aligned using Multiple Sequence Comparison by Log-Expectation (MUSCLE) as implemented in MEGA X version 11.0.13 software (36). All putatively functional LILRs found in the studied Carnivora were included, along with the human LILRs and representatives of the bovine and caprine group 1 and group 2 LILRs (21). The *novel Ig-like gene* was included for the species in which it is putatively functional. Phylogenetic trees were constructed using the Neighbor-Joining method, the Tamura-3 parameter model for nucleotide substitutions, and pairwise deletion of ambiguous positions within MEGA X. Node support was tested by 1,000 bootstrap iterations. The analysis involved 150 nucleotide sequences and there were a total of 2504 positions in the final dataset. The list of CDS sequences used is available as **Supplementary Data 1**. The predicted amino acid sequences of the LILRs were also aligned by MUSCLE and trees were generated by the Neighbor-Joining method in MEGA X, using the p-distance method for evolutionary distances. All ambiguous positions were removed for each sequence pair. The analysis involved 150 amino acid sequences with a total of 762 positions in the final dataset. The relationships of individual Ig domains isolated in BioEdit version 7.2.6 based on NCBI´s Conserved Domain Search (https://www.ncbi.nlm.nih.gov/Structure/cdd/wrpsb.cgi) were tested in the same manner.

## 3 Results

### 3.1 Felidae

**Figure 1:**
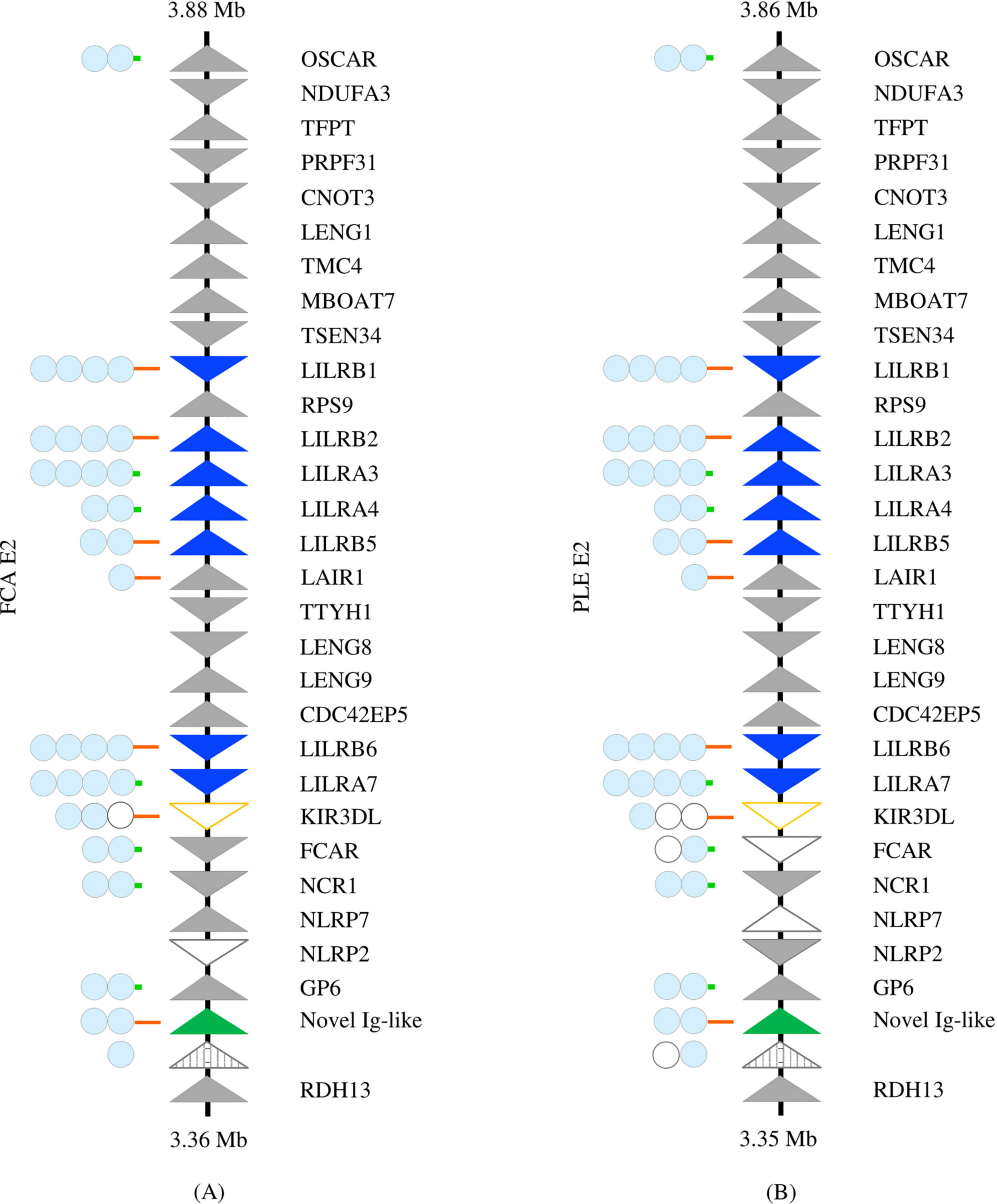
Comparison of the Leukocyte Receptor Complex between the Felinae and Pantherinae. The LRC of **(A)** *Felis catus* (NC_058382.1) and **(B)** *Panthera leo* (NC_056693.1), as representatives of the sub-families, is depicted. Solid triangles represent putatively functional genes, open triangles represent pseudogenes, and striped triangles represent gene fragments. LILRs are highlighted in blue, KIR in orange, and the *novel Ig-like gene* in green. Genes comprising Ig domains are schematized to the left. Light blue circles indicate intact Ig domains, while open circles represent disrupted Ig domains. Long orange lines represent cytoplasmic tails with functional ITIMs; short green lines represent the cytoplasmic domains of activating receptors.

The Felidae LRC is located on chromosome E2 in all chromosome-level SMLR felid assemblies except the Sunda clouded leopard, where it is found on provisional chromosome 17. The overall genomic organization of the region is conserved within the family and is illustrated in **Figure 1**. Deviations in the gene content of the SMLR chromosome-level assemblies, excluding the LILRs, are listed in **Table 1**. Although *NLRP2*, *NLRP7*, *GP6*, and the *novel Ig-like gene* (all of which are located adjacent to each other) are missing in the Canada lynx assembly, *GP6* was identified by a BLAST of the whole genome shotgun (WGS) sequence, and the status of the other genes in this block may be in question.

**Table 1:** Variations on the gene and pseudogene content in the non-LILR genes of the LRC in the ten assessed Felidae assemblies.

Species	Putative pseudogenes	Missing genes
*Acinonyx jubatus*	*KIR3DL*, NCR1*	–
*Felis catus*	*KIR3DL*, NCR1*, *NLRP2*	–
*Felis chaus*	*KIR3DL*	–
*Leopardus geoffroyi*	*FCAR*, *KIR3DL*	–
*Lynx canadensis*	*CNOT3*, *KIR3DL*, *LAIR1*, NCR1*	*NLRP2*, *NLRP7*, GP6**
*Neofelis nebulosa*	*KIR3DL*, NCR1*, *NLRP2*, *NLRP7*, *OSCAR*	–
*Panthera leo*	*FCAR*, *KIR3DL*, NCR1*, *NLRP7*	–
*Panthera tigris*	*FCAR*, *KIR3DL*, NCR1*	–
*Prionailurus bengalensis*	*KIR3DL*, *NCR*1*, *NLRP7*	–
*Prionailurus viverrinus*	*KIR3DL*, NCR1*, *NLRP7*	–

*NCR1 appears to be pseudogenized in the reference assemblies of these species but has been shown to be functional (37). **In *Lynx canadensis*, *GP6* was found in the WGS but not in the reference assembly.

The structure of the LRC was also preserved in the short-read and/or scaffold-level Felidae assemblies for which this region was contained on a single scaffold (the black-footed cat, the bobcat, the cougar, the jaguar, the leopard, the manul, and the snow leopard). The LRC is fragmented in the caracal, the Iberian lynx, the jaguar, and the yagouaroundi assemblies, but it was possible to reconstruct the region across a series of scaffolds. Although the proper order of the scaffolds could not be confirmed, these reconstructions were consistent with the organization of the LRC seen in other felids. The LRC was too fragmented to reconstruct for the Iriomote cat and the Sunda clouded leopard.

**Figure 2:**
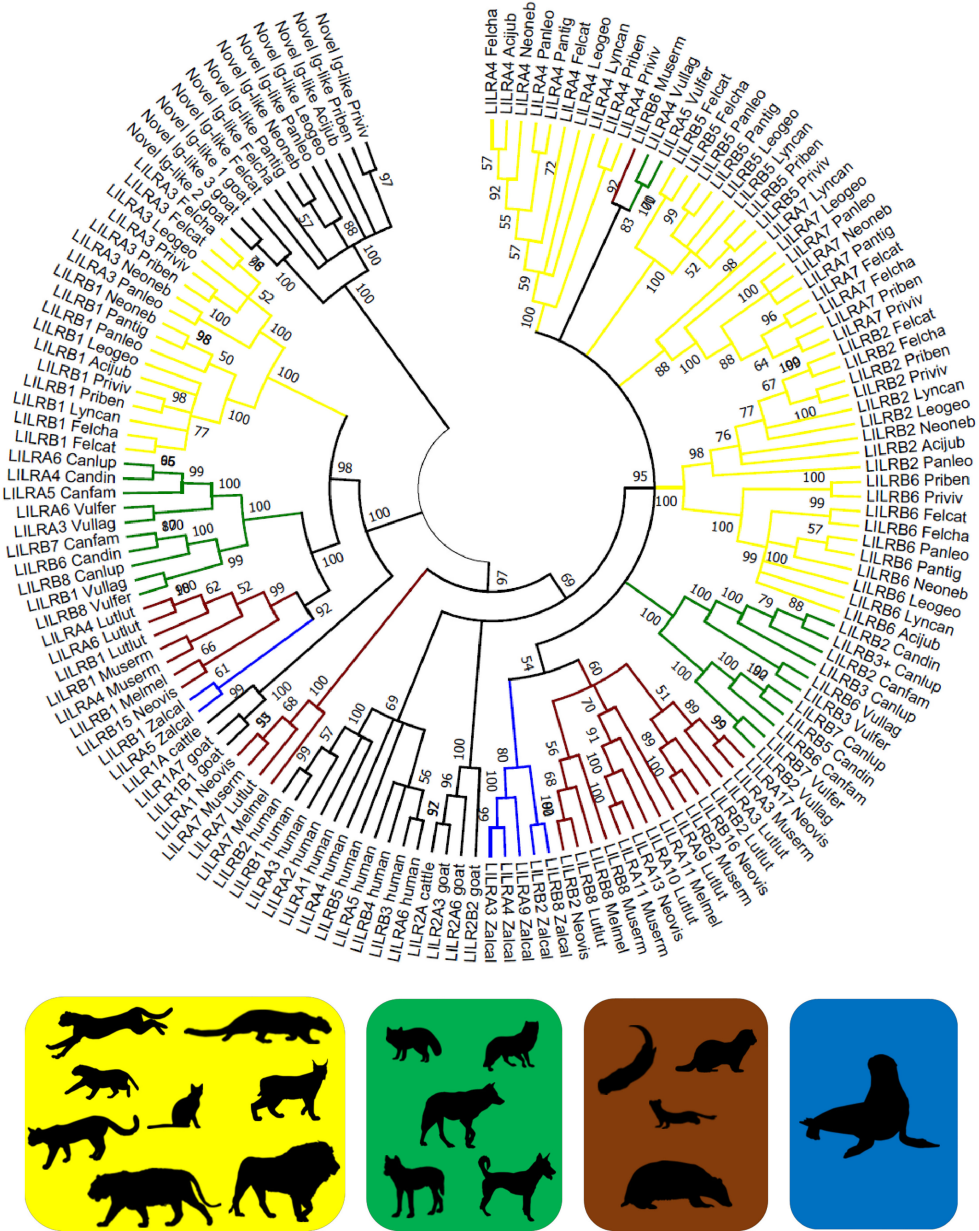
Phylogeny of putatively functional LILR genes in Carnivora. Coding sequences were compared to bovine, caprine, and human LILRs (**Supplementary Data 1**) by the Neighbor-Joining method and Tamura 3-parameter model in MEGA X. A bootstrap consensus tree is presented with branches reproduced in over 50% of 1000 replicates. The tree was rooted to the *novel Ig-like gene* sequences. Four Carnivora families are highlighted: Felidae (*yellow*) – the domestic cat (Felcat), jungle cat (Felcha), Bengal cat (Priben), fishing cat (Priviv), cheetah (Acijub), Geoffroy’s cat (Leogeo), Canada lynx (Lyncan), clouded leopard (Neoneb), lion (Panleo), tiger (Pantig); Canidae (*green*) – wolf (Canlup), domestic dog (Canfam), dingo (Candin), Tibetan sand fox (Vulfer), arctic fox (Vullag); Mustelidae (*brown*) – ermine (Muserm), European badger (Melmel), American mink (Neovis), Eurasian otter (Lutlut); and Otariidae (*blue*) – California sea lion (Zalcal). Silhouettes were adopted from PhyloPic and are credited at https://www.phylopic.org/permalinks/421bad89be8cca7302c414998c6370821df1c98c3db161ddbcd1d88c8df8a7a8.

All ten of the felid assemblies meeting the inclusion criteria contain seven syntenic LILR genes or pseudogenes (**Table 2**; **Supplementary Figure 1**). Each of these genes was confirmed to be highly homologous across species by phylogenetic analysis (**Figure 2**). The majority are located in two clusters within the LRC. *LILRB2*, *LILRA3*, *LILRA4*, and *LILRB5* are found together between *RPS9* and *LAIR1* and share their orientation. *LILRB1* is separated from this cluster by *RPS9* and is oriented in the opposite direction. *LILRB6* and *LILRA7* are found together between *CDC42EP5* and a *KIR3DL* pseudogene and again share their orientation (**Supplementary Figure 2**). *KIR3DL* is a pseudogene in all analyzed felid genomes.

**Table 2:** LILR gene content of Felidae assemblies.

Species	*Acinonyx jubatus*	*Felis catus*	*Felis chaus*	*Leopardus geoffroyi*	*Lynx canadensis*	*Neofelis nebulosa*	*Panthera leo*	*Panthera tigris*	*Prionailurus bengalensis*	*Prionailurus viverrinus*
LILRA 4-Ig putatively functional/pseudogenes	0/2	2/0	2/0	2/0	1/1*	2/0	2/0	1/1	2/0	2/0
LILRB 4-Ig putatively functional/pseudogenes	3/0	3/0	3/0	3/0	3/0	3/0	3/0	2/1	3/0	3/0
LILRA 2-Ig putatively functional/pseudogenes	1/0	1/0	1/0	1/0	1/0	1/0	1/0	1/0	1/0	1/0
LILRB 2-Ig putatively functional/pseudogenes	0/1	1/0	1/0	1/0	1/0	0/1	1/0	1/0	1/0	1/0
Total putatively functional LILRs/pseudogenes	4/3	7/0	7/0	7/0	6/1	6/1	7/0	5/2	7/0	7/0
*Novel Ig-like* between *GP6* and *RDH13*/pseudogenes/fragments	1/0/1	1/0/1	1/0/1	1/0/1	0/0/0	1/0/1	1/0/1	1/0/1	1/0/1	1/0/1

*In the Canada lynx, *LILRA7* contains two deletions relative to the *F. catus* reference mRNA such that the ORF is disrupted and restored with 3 amino acids affected. Due to the quick restoration of the ORF, this gene is considered putatively functional.

In the felid species for which only short-read or scaffold-level assemblies were available, evaluation of the LILRs was only possible when breaks between scaffolds did not interrupt the LILR clusters, which precluded evaluation of the caracal, the Iberian lynx, the Iriomote cat, the Sunda clouded leopard, and the yagouaroundi. In the bobcat and leopard (SMLR scaffold-level assemblies) as well as in the snow leopard (linked short reads, chromosome level), the same seven LILRs described above were found, and all are putatively functional. In the manul (Oxford Nanopore [ONT], scaffold level), these seven LILRs were identified but only two are putatively functional. In the cougar (combination short-read and ONT, scaffold level), only four LILRs (two functional) could be identified alongside many gene fragments. In both chromosome-level short-read assemblies, the black-footed cat and the jaguar, fewer LILRs were identified, with *LILRB6* and *LILRB7* found in the black-footed cat and *LILRA4* and *LILRB5* found in the jaguar. However, *LILRB1* and *LILRA3* were identified by Splign in an older jaguar assembly (GCA_004023805.1). A comprehensive picture of the LILR sub-region of the LRC (from *LILRB1* to *KIR3DL*, inclusive) was generated in VISTA comparing the ten SMLR chromosome-level felid assemblies (**Supplementary Figure 2**). The differences between species are minimal. A second VISTA comparison of nine felid assemblies not meeting these criteria to the domestic cat assembly shows more differences, including rearrangements and gaps, especially in the short-read assemblies. Nonetheless, the overall gene content is still similar across species. (**Supplementary Figure 3**).

In contrast to the other felid LILRs, *LILRA7* spans 6 Ig-like domains identified by BLAST in all of the SMLR Felidae assemblies except the cheetah and the Canada lynx. The 5^th^ and 6^th^ Ig groups contain missense or nonsense mutations in many species. In some cases, the intron/exon splice sites are disrupted, whereas in others they are intact at both ends of the exon. The functional significance of this variation remains unknown and the expression status of *LILRA7* needs verification.

In all felids except the Canada lynx, an additional putatively functional 2-Ig inhibitory gene was found between *GP6* and *RDH13*, along with a gene fragment containing Ig-like domains. No other gene fragments containing Ig-like domains were identified in the LRC of any Felidae species.

### 3.2 Other Feliformia

Other Feliformia encompassed representatives of the Herpestidae and Hyaenidae families. Evaluation of the LILR content of short-read assemblies was limited by assembly quality and poor resolution of the LILR clusters. Nonetheless, it was possible to reconstruct the LRC across five or fewer scaffolds in four species: the meercat, the striped hyena, the aardwolf, and the spotted hyena. No significant deviations from the Felidae LRC structure or LILR gene content were identified in these species. Notably, the aardwolf and the striped hyena both appear to possess a LILR gene syntenic to felid *LILRA7* that also spans six Ig domain exons. In contrast to felid *LILRA7*, however, both of these genes possess long cytoplasmic tails with intact ITIMs, and therefore are presumably inhibitory receptors. In both species, all six Ig domains of the presumed receptor and the relevant splice sites appear intact.

### 3.3 Canidae

The LRC was found on chromosome 2 in the arctic fox, chromosome 4 in the Tibetan sand fox, and chromosome 1 in the wolf, the domestic dog, and the dingo. The overall genomic organization of the region is conserved across the canid species (**Supplementary Figure 4**) and is very similar to the felids. Consistent differences in the framing genes between the two families are the absence of *NLRP7* in the Canidae and the higher frequency of pseudogenization of *FCAR* and *LENG9* in the canids. No KIR gene or pseudogene was identified in any canid. Four (dingo, dog) or five (wolf, both fox spp.) putatively functional LILR genes were found (**Table 3**). These include three 4-Ig domain *LILRB* genes and one 4-Ig *LILRA* which are syntenic and homologous across the five (sub)species. An additional *LILRA* with 2 Ig groups is putatively functional in the foxes, whereas a frameshift mutation in the *Canis* subspecies produces a premature stop codon. An additional 4-Ig *LILRA* pseudogene was found in all assemblies except the dingo. The situation in the wolf is specific in that the cluster of genes from *LILRB3* to *FCAR* appears to be duplicated. Thus, the wolf assembly contains one more functional 4-Ig *LILRB* relative to the other canids. For the purposes of this article, the duplicated genes and pseudogenes are denoted with a plus superscript (e.g., *LILRB3^+^*).

**Table 3:** LILR gene content of the LRC in Canidae assemblies.

Species	*Vulpes lagopus*	*Vulpes ferrilata*	*Canis lupus familiaris*	*Canis lupus dingo*	Canis lupus*
LILRA 4-Ig putatively functional/pseudogenes	1/2	1/2	1/2	1/1	1/3 (1/2)
LILRB 4-Ig putatively functional/pseudogenes	3/1	3/1	3/0	3/0	4/2 (3/1)
LILRA 2-Ig putatively functional/pseudogenes	1/0	1/0	0/1	0/1	0/1 (0/1)
Total putatively functional LILRs/pseudogenes	5/3	5/3	4/3	4/2	5/6 (4/4)
*Novel Ig-like* putatively functional/pseudogenes/fragments located between *GP6* and *RDH13*	0/0/1	0/0/1	0/0/1	0/1/0	0/1/0 (0/1/0)
Other fragments containing Ig-like domains	1	1	1	2	7 (5)
Ig domain loci without signal peptide	4	4	1	3	2 (1)

*The numbers in parentheses represent the count if the duplicated segment of the wolf LRC is discounted as an artifact.

A fragment of the *novel Ig-like gene* described in the Felidae was found between *RDH13* and *GP6* by Splign using the domestic cat gene as a query. This locus constitutes a full-length pseudogene in the dingo and the wolf but does not appear to be functional in any of the canids.

### 3.4 Mustelidae

In the Mustelidae, the LRC is located on chromosome 19 of the ermine and the European badger, chromosome 17 of the Eurasian otter, and chromosome 7 of the American mink. The LRC is contiguous and conforms to the typical genomic architecture described for felids with the notable exception of the American mink, in which it is divided between two regions separated by approximately 15 megabases (**Supplementary Figure 5**). Each of the two regions comprises roughly half of the LRC gene count, and the arrangement of genes within each region is consistent with that found in other species. *LENG8*, *LENG9*, *CDC42EP5*, and *FCAR* were not found in the American mink assembly or in the WGS by BLAST or Splign.

Exploration of the annotated genes on American mink chromosome 7 and comparison to the positions of these genes in the European badger assembly demonstrated the rearrangement of large blocks of genes in the regions neighboring the LRC in the American mink assembly and the fusion of European badger chromosomes 8 and 19 into American mink chromosome 7.

Four (European badger) to nine (Eurasian otter) putatively functional LILR genes were identified in the Mustelidae, along with three (Eurasian otter) to 17 (American mink) pseudogenes (**Table 4**). In many cases, these genes are syntenic and homologous (based on phylogenetic analysis) across several or all four of the mustelid species studied. However, because of the expansion and variability of the LILR family in the Mustelidae, it was not possible to determine the relationships between the pseudogenes of different species with the same clarity as in the Felidae.

**Table 4:** LILR gene content of Mustelidae LRCs.

Species	*Mustela erminea*	*Meles meles*	*Neogale vison*	*Lutra lutra*
LILRA 4-Ig putatively functional/pseudogenes	4/4	2/6	3/10	6/1
LILRB 4-Ig putatively functional/pseudogenes	3/2	2/2	3/3	3/0
LILRA 3-Ig putatively functional/pseudogenes	0/0	0/1	0/0	0/0
LILRB 3-Ig putatively functional/pseudogenes	0/0	0/1	0/0	0/0
LILRA 2-Ig putatively functional/pseudogenes	0/0	0/0	0/2	0/0
LILRB 2-Ig putatively functional/pseudogenes	1/0	0/1	0/2	0/1
LILR pseudogenes with neither activating nor inhibitory domains	1	0	0	1
Total LILRs putatively functional/pseudogenes	8/7	4/11	6/17	9/3
Fragments containing Ig-like domains	1	2	3	2
Ig domain loci without signal peptide	4	5	4	2

As a minor deviation in the structure of the LRC, in the Eurasian otter, a *LAIR1* pseudogene is located in the midst of the LILR cluster ranging from *TTYH1* to *RPS9*, rather than in its typical position adjacent to *TTYH1*, and thus divides this cluster of LILRs into two groups.

Several points should be noted regarding the classification of specific mustelid LILRs. The 2-Ig LILR gene found in the ermine, *LILRB6*, has been classified as putatively functional but codes for a long cytoplasmic tail with both ITIMs mutated (nonfunctional). Therefore, the expression of LILRB6 and its functions remains questionable. Based on alignments in Splign, homologs to ermine *LILRB6* were identified in the Eurasian otter, the European badger (one such pseudogene each), and the American mink (two pseudogenes), but are clearly pseudogenized due to disruptions in their Ig domains. The American mink *LILRA3* product similarly lacks a charged residue in the predicted TM domain. In addition, Eurasian otter *LILR_12* and ermine *LILR_14* lack the TM domain and tail. Therefore, they have been assigned neither activating nor inhibitory function and are classified as pseudogenes. However, it is possible that one or both encode a secreted product with some function.

No Ig groups were identified by BLAST between *GP6* and *RDH13* in any Mustelidae species, and Splign using the domestic cat *novel Ig-like gene* from this locus as a query produced no hits in this family.

### 3.5 Otariidae

The LRC of the California sea lion is located on chromosome 17. It conforms to the genomic architecture described for other carnivores. It contains seven putatively functional LILRs, four 4-Ig *LILRA*s and three 4-Ig *LILRB*s, and two LILR pseudogenes, a 4-Ig *LILRA* and a 2-Ig *LILRAB* containing both activating and inhibitory motifs (**Supplementary Figure 6**). A gene fragment comprising a signal peptide and a single Ig-like domain was identified at the typical locus of the *novel Ig-like gene* in felids between *GP6* and *RDH13*, but no alignment with this gene was found on Splign. Two other Ig-like domains not associated with SPs were identified elsewhere in the LRC. Thus, while no consistent differences were found across carnivore families in terms of the structure of the LRC, the LILR content of the California sea lion more closely resembles that of the Felidae and Canidae than the Mustelidae, despite the fact that out of the three, the Mustelids are the most closely related family to the sea lion in evolutionary terms.

### 3.6 Phylogenetic analysis of LILRs

Phylogenetic analysis produced similar results regardless of whether coding sequences (**Figure 2**) or amino acid sequences (**Supplementary Figure 7**) were used as the basis of the analysis. The branching structure of the tree was determined primarily by the clustering of orthologous LILRs within families. In most cases the evolutionary relationships between and within Carnivora families were reproduced, with genuses, families, and the suborders Caniformia and Feliformia forming clusters at progressively higher levels. However, in the cases of *LILRA4* and *LILRB6*, the *Felis* spp. clustered more closely with the Pantherinae than with the *Prionailurus* spp.

Several bovine, caprine, and human LILRs were included in the phylogenetic analysis to assess whether the Carnivora LILRs are similarly divided into group 1 and group 2 genes. Felid *LILRA3* and *LILRB1* and their homologues in other Carnivora spp. clustered with Cetartiodactyla group 1 genes to form one main branch, while the remaining Carnivora LILRs clustered with the human and Cetartiodactyla group 2 genes to form a second branch, thus largely reflecting the same division into two lineages. However, some of Mustelidae LILRs fell between these two groups. Phylogenetic analysis of the amino acid sequences of the Ig domains of these receptors suggests that the first Ig domain is closely related to the group 1 LILRs, while the following 3 Ig domains are characteristic of the group 2 LILRs (**Supplementary Figure 8**).

Based on phylogenetic analysis of the 6-Ig domain receptors, artificially constructed felid LILRA7 shows a duplication of the third and fourth Ig groups, in contrast to the duplication of the first and second Ig groups seen in the mouse and cow (**Supplementary Figure 9**). The same applies for the putatively functional 6-Ig LILRB receptor found in the aardwolf and the striped hyena.

## 4 Discussion

Until recently, comparative studies of the LILRs have been limited in part by the technical difficulty of sequencing the LRC, which contains a number of closely related genes and is highly repetitive. Such regions are generally difficult to assemble from short-read sequencing data (38, 39), and therefore the LRC is often fragmented or misassembled in short-read assemblies. In scaffold-level assemblies, breaks are especially common within the LILR clusters, and portions of these genes may also be found on small unplaced scaffolds. Long-read assemblies are better able to map such technically challenging regions without breaks; the impact of this method has been such that it was named “Method of the Year 2022” by *Nature Methods* (40). For these reasons, this study focused on long-read chromosome-level assemblies. However, basecalling errors are more frequent in long-read assemblies (41), despite reported improvements in basecalling for both SMLR (42) and ONT sequencing (43). Thus, some genes with only minor changes in their sequence (1-2 bp indels/mutations) may be erroneously categorized as pseudogenes and in fact may be functional.

The LRC is highly conserved across the ten felid assemblies meeting the inclusion criteria (**Supplementary Figure 1**) and it is identical in another *F. catus* genome assembly (GCA_016509815.2). Moreover, no inconsistencies in the content of the LILR sub-region (**Supplementary Figure 2**) were identified in the short-read or scaffold-level Felidae assemblies (**Supplementary Figure 3**), nor in the short-read assemblies of other Feliformia species (data not shown).

Within the Carnivora more largely, chromosome-level long-read assemblies were available for several species of both the Canidae and Mustelidae families, allowing the intrafamily variability of the LRC to be assessed and compared with the Felidae. Because these assemblies met the same inclusion criteria as the focal felid assemblies, concerns that apparent variability may be the result of different sequencing technologies are reduced. In addition to these two families, a long-read chromosome-level assembly was available for the California sea lion; this is the only other assembly of a Carnivora species meeting these criteria, and it has been included to broaden the view of the order. The overall architecture of the LRC was again consistent across species, suggesting that the conserved structure of the LRC observed in the Felidae is typical for the order Carnivora. However, two partial exceptions should be noted: the wolf and the American mink.

In the wolf, the block of genes spanning from *FCAR* to *LILRB3* appears to be duplicated. Although the sequences of this region and its duplicate are very similar, they are not identical. The cognate pairs of the (pseudo)genes often contain 2 to 3 substitutions per gene and differ in length by up to 52 base pairs. *LILRB3* and *LILRB3^+^*, the only pair of putatively functional LILRs resulting from the duplication, differ sufficiently in their sequences that on phylogenetic analysis *LILRB3^+^* clustered more closely with the orthologous gene in the dingo and the dog than with wolf *LILRB3*. However, whether this duplication is a true feature of the wolf genome or an artifact of the assembly remains to be determined.

The apparent division of the LRC in the American mink into two subregions separated by approximately 15 megabases represents a significant divergence. Previous research on the phylogeny of the LRC has not, to the best of the authors’ knowledge, described such a division in any other mammalian species (2). Nonetheless, the fusion of two chromosomes which are separate in many Mustelidae karyotypes (including European mink, forest polecat, lesser weasel, mountain weasel, Japanese sable, striped polecat, and European badger) into the single American mink chromosome 7 has been elucidated by G-banding and FISH data (44). The genes present on the respective chromosomes in the examined assemblies confirm the fusion of *M. meles* chromosomes 8 and 19 into *N. vison* chromosome 7. Graphodatsky et al. (44) propose an ancestral Carnivora karyotype (ACK), with ACK chromosome 19 corresponding to chromosome E2 in the Felidae and to the p arm of *N. vison* chromosome 7, which corresponds with the locations of the LRC in these species, and confirming the homology described in an earlier FISH study (45). Thus, it may be that during the karyotype evolution that produced *N. vison* chromosome 7, chromosome breakage and reassembly split the LRC into two regions. Unfortunately, no other assemblies of *Neogale* spp. are available for comparison; the only other assembly available for the genus is a second *N. vison* genome, which is assembled only to the scaffold level and is insufficient to confirm or disprove the division of the LRC. The closest relatives for which assemblies are available are the *Mustela* spp.; however, the chromosomal rearrangement affecting *N. vison* chromosome 7 did not occur in the Mustelae (44). This two-part arrangement of the American mink LRC could allow for the generation of additional diversity in the LILRs as they would be less likely to be inherited en bloc. In this context, it is interesting that the mink possesses the largest repertoire of LILR (pseudo)genes of any of the studied Mustelidae. This possible difference in the genomic organization of the LRC between closely related species demonstrates the importance of studies conducted at the species level in rapidly evolving regions such as the LRC.

The SMLR felid assemblies each contain seven orthologous LILRs. Although we hypothesized that there could be differences in the LILR content between the Felinae and Pantherinae, no such differences were identified. The preserved localization of gene components (introns, exons, UTRs) in the VISTA graphic further attests to the conserved nature of the LILR family in Felidae (**Supplementary Figure 2**). In the case of the Canada lynx, pseudogenes should be considered in light of the identified anomalies in the assembly. Similarly, the clouded leopard assembly was recently made available and is not yet expected to be in its final form. In the tiger, some pseudogenes again may be an artifact or the result of interindividual variability, as *LILRA3* is putatively functional in a recently released assembly based on linked short reads and using Hi-C scaffolding (GCA_024034525.1) (46). The individual used for the SMLR reference assembly was a cross-species hybrid (tiger X lion), and it is possible that the challenge of separating the two sequences led to some misassembly in technically challenging regions like the LRC. The cheetah, on the other hand, has the most pseudogenes (three), and interestingly the cheetah immune system is known to have several specific features. The variation in the cheetah MHC has traditionally been considered to be notably low (47). While much higher genetic diversity within MHC I (48) and MHC II (49) loci has been described recently, cheetahs still show MHC II-DRB diversities lower than other large felids like Bengal tigers (50), Eurasian lynx (51), and leopards (52). Cheetahs seem to achieve immunocompetence through higher constitutive innate immunity but lower induced innate and adaptive immunity compared to a sympatric leopard population (5). It is tempting to hypothesize that the higher number of LILR pseudogenes in this species could be related to these specificities, but focused research of LILR expression and function is needed to draw any conclusions.

Differences in the LILR gene content of the Felid assemblies not meeting the inclusion criteria may to some extent reflect the sequencing technology. In the bobcat and the leopard, the two SMLR scaffold-level assemblies, orthologues of the seven described LILRs were identified. This was also the case for the snow leopard assembly, which was generated by linked short reads. The manul assembly is also a scaffold-level long-read assembly but based on ONT rather than SMLR, and it also contains these seven orthologues, although only two appear to be functional. The cougar assembly, generated by a combination of short-reads and ONT, contains many LILR fragments but only two full genes, while the two short-read assemblies with the LRC contained on a single scaffold, the black-footed cat and the jaguar, each contain two LILRs and no fragments within the LRC. However, the number of Ig domains identified in a BLAST of the WGS in both cases far exceeded the number of such domains seen in the LRC along with the X chromosome, and in both cases was similar to the number of Ig domains found by BLAST of the WGS in the domestic cat. This suggests that in these assemblies some LILRs may be found on small unplaced scaffolds. This correlation of the number of identified LILRs with the use of different sequencing technologies suggests that long-reads are indeed more successful at resolving the LILR subregions.

Regarding the Carnivora more broadly, the overall LILR content is similar between the Felidae (seven LILRs), the Canidae (six to eight), and *Z. californianus* (nine). A possible exception is the wolf, which may have a total of 11 LILRs if the duplication described is a true feature vs. eight if it is taken as an artifact of the assembly. The Mustelidae possess more LILR genes, ranging from 12 (Eurasian otter) to 23 (American mink) in the species studied. The number of LILR fragments is also higher in the Mustelidae. However, this does not translate into a similarly higher number of functional LILRs in the mustelids: on average they possess 6.75 putatively functional LILRs, compared to 6.3 in the felids. Indeed, Felidae possess a notably higher ratio of functional LILRs to pseudogenes (90.00%) compared to the Mustelidae (41.54%) or the Canidae (57.50%; 59.46% with exclusion of the wolf duplication). Due to the so far poor characterization of LILR functions, it is difficult to make a link between interspecific variation in the LRC gene count and potential selective pressures leading to such a differentiation.

The intrafamily variability in the LILR complement was lowest in the Felidae. The other end of the spectrum is again represented by the Mustelidae, which show the largest variability between species in terms of the total number of LILR loci, the total number of putatively functional genes, and the ratio of putatively functional to pseudogenized LILRs.

The significance of the enlarged family of LILRs and its interspecies variability in the mustelids remains to be elucidated. One hypothesis may be that in the Mustelidae the LILRs fulfill the function of an expanded NK cell receptor family binding MHC class I ligands, similarly to the KIR in humans (53) and KLRA in mice (54). In this interpretation, many genes and pseudogenes could be the result of changing pathogen pressures leading to the rapid evolution and inactivation of LILR genes. However, the LILRs bind the α3 and β2 microglobulin domains of MHC-I rather than the highly variable α1 and α2 domains where KIR and KLRA bind (24), and this may limit their capacity to fulfil a similar function, although they are known to be capable of distinguishing between self and non-self MHC-I in mice (29, 55). Alternatively, the expansion of the Mustelidae LILR family could be more a feature of the evolution of the chromosomal region than the result of selective pressures on the LILRs, and pseudogenes may have arisen as nonfunctional sequences through mechanisms such as the duplication of neighboring sequences.

A putatively functional *novel Ig-like gene* was found between *GP6* and *RDH13* in all studied felids except the Canada lynx (caveat again, some misassembly in this part of the Lynx assembly is suspected), as well as in the short-read or scaffold-level Felidae assemblies. A pseudogene or fragment homologous and syntenic to this gene was identified in all studied Feliformia and Canidae species. Comparably, only a signal peptide and Ig-like group was found at this locus in the Californian sea lion. No Ig-like gene was found here in any of the Mustelidae species. Thus, the Felidae are so far the only Carnivora family identified carrying a putatively functional copy of this gene.

Phylogenetic analysis suggests that this gene is related to the *novel Ig-like* genes recently identified in pigs and subsequently in other species including cattle, sheep, goats, horses (20) and camels (56). The gene content of this Ig-like family varies from one (camels) to seven (cattle) genes. In the previously described cases, these genes are located between *NLRP2* and *NLRP7*, which differs slightly from the locus identified in Felidae. No genes or immunoglobulin domain coding sequences were found in any felid between *NLRP2* and *NLRP7* in our study.

Previously published studies characterizing the LRC and the LILRs for multiple species within a family are limited. Those which can serve for comparison to the Felidae are the Hominidae, represented by humans, chimpanzees, bonobos, gorillas, and orangutans (18); the Bovidae, represented by cattle (*Bos taurus*) (22, 57) and goats (*Capra hircus*) (21); and the Camelidae, represented by the Bactrian camel (*Camelus bactrianus*), the dromedary (*Camelus dromedarius*), and the wild camel (*Camelus ferus*) (56).

The complement of genes in the LRC in each of these families is similar to that described for the Felidae, but the relative orientation of some blocks of framing genes differs in the Bovidae, and in some places differs even between cattle and goats (21, 22). The architecture of the LRC in the Old World camels is most similar to that of goats, with the block of genes from *LAIR1* to *CDC42EP5* inverted in comparison with the Carnivora. In all cases the LILRs remain divided into two clusters.

The LILR complement in the Hominidae and the Bovidae appears to be larger and more variable than in the Felidae. The Hominidae possess 13 (human, gorilla, orangutan) or 14 (chimpanzee, bonobo) LILRs each, of which seven (gorilla) to 12 (chimpanzee) are putatively functional (18). Relative to the Felidae, this is a larger number of both total and putatively functional LILRs, as well as greater intrafamily diversity in the number of LILRs and the proportion of functional genes. In the Bovidae, sixteen LILRs and the closely related *FCG2R* gene have been described in cattle (22), comprising five activating and seven inhibitory receptors (70.6% potentially functional genes) while five soluble forms may represent pseudogenes. Eight LILRs and *FCG2R* have been described in goats, of which four are activating receptors and two are inhibitory (66.7% putatively functional genes) (21). Comparing just these two species shows higher variability in the LILR complement of the Bovidae than was found in the Felidae.

The Camelidae LILR family is comparable to that of the Felidae, with six (Bactrian camel, dromedary) or seven (wild camel) LILRs present in each species. These are all putatively functional in the SMLR assembly of the wild camel, while only three (*C. dromedarius*) or four (*C. bactrianus*) are functional based on targeted resequencing in the domestic species (56). This is one of the most similar families to the Felidae in terms of the LILR gene content, and it is notable that more closely related families both to the Felidae (e.g., the Mustelidae) and to the Camelidae (e.g., the Bovidae) are more divergent in their complement of LILRs. Thus, the analysis of an evolutionarily close species is not a reliable predictor of the LILR complement in an unstudied species.

Importantly, the methodology employed in the identification of LILRs may impact study findings. A comparison of the LILR gene content across 48 mammalian species was carried out using OrthoFinder augmented by a reciprocal best BLASTn hit search (3). A smaller complement of LILRs was reported for each of the three Carnivora species in that study than is presented here. Discrepancies also exist between the Hilton study and other studies discussed above, including differences in the number of LILRs identified in each of the higher primates (18) and in goats (21). In most cases, Hilton et al. counted, based on the automated annotations, fewer LILRs than other studies of the same species. These annotations are often inaccurate for tandemly duplicated gene families. In a parallel to the identification of fewer LILRs in the short-read Felidae assemblies in this study, it may be that short-read assembly quality in the region of the LRC inhibited the identification of LILRs in some cases. In species for which a long-read assembly is not available, a BLAST search of the WGS followed by targeted resequencing could be carried out to augment the findings from short-read assemblies but may still overlook some genes, e.g., those split onto two contigs. This underscores the importance of assembly quality in assessing the LRC and particularly the LILRs, as well as the challenges of comparing between species and/or studies relying on different assembly technologies.

Several further steps may contribute to our understanding of the characteristics of the LILRs in Felidae and other families. Targeted resequencing of LILR genes to confirm their sequences and assess allelic variants would provide information about both the inter- and intraspecies variability of the LILRs and would allow subsequent selection analyses to identify sites under purifying and diversifying selection. In addition, expression studies would confirm the expression status of these genes in different tissue/cell types and may illuminate connections between their specificities in *A. jubatus* and known features of the cheetah immune system. Further, an expanded study of the LILR gene family in other Carnivora species, once long-read assemblies become available, would give a more complete picture of the order and confirm or repudiate the trends seen in the species sampled here. In light of the known differences in the LILR gene family of closely related species, such studies would be of value.

In conclusion, the genomic architecture of the LRC is highly conserved across the Felidae. This same overall structure is also largely conserved in the other studied carnivore species, with the major exception of *N. vison*, in which a chromosomal rearrangement appears to have split the LRC into two separate regions. There also appears to be a duplication of a portion of the LRC in *C. lupus*. The organization of the LRC is similar in the more distant Hominidae, Bovidae, and Camelidae families, with the caveat that one or more blocks of genes appear to be inverted relative to their position in the Carnivora LRC. The LILR gene content is also conserved within the Felidae, with a total of 7 orthologous (pseudo)genes identified in each species, all of which are functional in most studied felids. This is similar to the number of LILRs found in the Canidae (6 to 11) but lower than that found in the Mustelidae (12 to 23), and the percentage of pseudogenes is higher in both of these families. Among the studied carnivores, the variability of the LILR gene content between species of a family is lowest in the Felidae and highest in the Mustelidae. Overall, the phylogenetic tree of LILRs reflected the evolutionary relationships among the species, although there were some minor exceptions within the Felidae. Within the carnivores, presumed LILR orthologues clustered together more closely than with other LILRs from the same species but only within families. Two lineages of LILRs were identified in the Carnivora as previously described in Cetartiodactyla. A gene that clustered with the *novel Ig-like gene* described by Schwartz and Hammond (20) was also identified but was putatively functional only in the Felidae. In comparison to more distant families, the total number of LILRs is higher in the Hominidae, similar in the Camelidae, and varies significantly between species in the Bovidae. The within-family variability in the LILR complement is larger in the Hominidae and the Bovidae compared to the Felidae and is similar in the Camelidae. This indicates that evolutionary closeness is not a good predictor of similarity in the LILR gene family, potentially due to the action of selective pressures and the evolutionary flexibility of the LRC. As high quality long-read assemblies become available for more species, the characterization of their LRCs will increase our knowledge of the interspecies variability of this region and the LILR gene family. Significant gaps remain in our knowledge of LILRs, and comparative studies may improve our understanding of these important genes in humans as well as informing our understanding of the phylogeny of the immune system and potentially offering targets for clinical diagnostics or treatment in the studied species.

## Data availability statement

The original contributions presented in the study are included in the article/**Supplementary Material**. Further inquiries can be directed to the corresponding author.

## Author contributions

AJ: Searched the available genomes, mapped the LRCs, performed bioinformatic analyses, extracted CDS for phylogenetic analysis, and drafted and edited the manuscript. JF: Participated in the study design, cross-checked all sequences, performed phylogenetic analyses, and edited the manuscript. PB: Conceptualization and design of the project and edited the manuscript. PH: Conceptualization and design of the study, organized collaborations, and edited the manuscript. All authors contributed to the article and approved the submitted version.
