# Supplementary material for: Comparative genomics of the Leukocyte Receptor Complex in carnivores
Source: Front Immunol. 2023 May 10;14:1197687. doi: 10.3389/fimmu.2023.1197687 (PMC10206138; doi:10.3389/fimmu.2023.1197687)
Supplement: Supplementary file 2 [file DataSheet_2.docx]

***Supplementary Material***

**1. Supplementary Data**

**Supplementary Data 1.** Nucleotide sequences of the coding regions of LILR genes and the novel Ig-like gene used for phylogenetic analysis (FASTA). Carnivore gene sequences were extracted from NCBI´s reference genomes (see Methods); bovine and caprine sequences were adopted from Schwartz et al. 2019; human sequences came from NCBI´s reference mRNAs: NM_006863.4 (LILRA1), NM_001130917.3 (LILRA2), NM_006865.4 (LILRA3), NM_012276.5 (LILRA4), NM_021250.4 (LILRA5), NM_024318.4 (LILRA6), NM_006669.7 (LILRB1), NM_005874.5 (LILRB2), NM_001081450.3 (LILRB3), NM_001278426.4 (LILRB4) and NM_001081442.3 (LILRB5).

**2. Supplementary Figures**

**Supplementary Figure 1.** Structure of the Leucocyte Receptor Complex (LRC) in Felidae. The gene sequence and putative functionality of genes is based on single-molecule long-read (SMLR) assemblies of the relevant chromosome (GenBank accession number) in the following species: (A) *Felis chaus* (CM034426.1), (B) *Panthera tigris* (NC_056674.1), (C) *Leopardus geoffroyi* (NC_059335.1), (D) *Neofelis nebulosa* (CM051615.1), (E) *Prionailurus viverrinus* (NC_062575.1), (F) *Prionailurus bengalensis* (NC_057352.1), (G) *Lynx canadensis* (NC_044317.1), and (H) *A. jubatus* (CM050188.1). Solid triangles represent putatively functional genes, open triangles represent pseudogenes, and striped triangles represent gene fragments. Rectangles represent Ig domain sequences not associated with a signal peptide or sequences for other receptor components. LILRs (*blue*), KIR (*yellow*), and the *novel Ig-like gene* (*green*) are highlighted. Genes comprising Ig domains are schematized to the left. Light blue circles indicate intact Ig domains, while open circles represent disrupted Ig domains. Long orange lines represent cytoplasmic tails with functional immunoreceptor tyrosine-based inhibitory motifs; short green lines represent the cytoplasmic domains of activating receptors with a positively charged residue in transmembrane domain.

**Supplementary Figure 2.** VISTA comparison of the LILR sub-region in SMLR assemblies of Felidae genomes. A plot of the Shuffle-LAGAN alignments of *Felis chaus*, *Prionailurus bengalensis*, *Prionailurus viverrinus*, *Leopardus geoffroyi*, *Lynx canadensis*, *Acinonyx jubatus*, *Panthera leo*, *Panthera tigris*, and *Neofelis nebulosa* to the *Felis catus* reference genome sequence is depicted. Conserved regions with more than 70% sequence similarity over a 100 base pair window are colored: non-coding sequences (*apricot*), exons (*purple*), untranslated regions (*cyan*). The LILR region was analyzed for the presence of long interspersed repeats (*red*), short interspersed repeats (*green*), long terminal repeats (*pink*), or different repeats (*yellow*) known from the cat genome, and genes (*dark gray arrows*) are annotated in the upper line. Contig (*light gray arrows*) rearrangements were limited and mostly occurred at repetitive sequences adjacent to *LAIR1* and *TTYH1*, respectively.

**Supplementary Figure 3.** VISTA comparison of the LILR sub-region in short-read or scaffold-level assemblies of Felidae genomes. A plot of the Shuffle-LAGAN alignments of *Lynx rufus*, *Panthera pardus*, *Otocolobus manual*, *Panthera uncia*, *Puma concolor*, *Felis nigripes*, *Panthera onca*, *Puma yagouaroundi*, and *Caracal caracal* to the *Felis catus* reference genome sequence is depicted. Color scheme applies as for Supplementary Figure 2 (see above). Contigs (*light gray arrows*) were commonly split and rearranged.

**Supplementary Figure 4.** Structure of the LRC in Canidae. The gene sequence and putative functionality of genes is based on SMLR chromosome-level assemblies of the following species: (A) *Vulpes lagopus* (NC_054825.1), (B) *Vulpes ferrilata* (CM044686.1), (C) *Canis lupus dingo* (NC_064243.1), (D) *Canis lupus familiaris* (NC_051805.1), and (E) *Canis lupus* (HG994383.1). The graphic schema used is the same as for Supplementary Figure 1 (see above). Cognate loci in the replicated region in the C. lupus assembly are indicated by a plus (*^+^*). In C. lupus, the cytoplasmic tail associated with LILRB2/LILRB2*^+^* is located 15 Kb from the Ig domain sequences.

**Supplementary Figure 5.** Structure of the LRC in Mustelidae. The gene sequence and putative functionality of genes is based on SMLR chromosome-level assemblies of the following species: (A) *Neogale vison* (NC_058097.1), (B) *Lutra lutra* (NC_062294.1), (C) *Mustela erminea* (NC_045632.1), and (D) *Meles meles* (NC_060084.1). The graphic schema used is the same as for Supplementary Figure 1 (see above). Long gray lines indicate long cytoplasmic tails with no functional immunoreceptor tyrosine-based inhibitory motifs.

**Supplementary Figure 6.** Structure of the LRC in the Californian sea lion. The gene sequence and putative functionality of genes is based on the available SMLR chromosome-level *Zalophus californianus* assembly (NC_045611.1). The graphic schema used is the same as for Supplementary Figure 1 (see above).

**Supplementary Figure 7.** Phylogeny of Carnivora LILR receptors based on amino acid sequences. Compared to Bovidae LILRs, they also come from two lineages of immunoglobulin-like domains and form group 1 (*magenta*) and group 2 (*violet*) receptors. Highlighted are families of Carnivora: Felidae (*yellow*), Canidae (*green*), Mustelidae (*brown*), and Otariidae (*blue*). The optimal Neighbor-Joining method tree with the sum of branch length = 9.14804286 is shown. The branch length scale represents the number of amino acid differences per site. Branch node values are given as the percentage of trees with the same clustering (1000 bootstrap replicates).

**Supplementary Figure 8.** Phylogeny of immunoglobulin-like domains of Mustelidae LILRA combined receptors. The amino acid sequences of individual Ig-like domains of the Eurasian otter (*Lutlut*), the European badger (*Melmel*), the ermine (*Muserm*), and the American mink (*Neovis*) receptor were compared to representatives of bovine group 1 (*magenta*) and group 2 (*violet*) Ig-like domains. The bootstrap consensus tree using the Neighbor-Joining method based on p-distances (MEGA X) is depicted with branch node values as the percentage of trees (out of 1000 replicates) in which the associated sequences clustered together.

**Supplementary Figure 9.** Phylogeny of LILR receptors with six immunoglobulin-like domains. Comparison of the amino acid sequences of individual Ig-like domains of the constructed Felidae receptors (*** - Ig-like domains with stop codons/frameshifts) with bovine and murine 6-Ig-domain receptors shows the difference in their organization. Four brackets of Ig-like domains are highlighted in different shades of blue. The bootstrap consensus tree using the Neighbor-Joining method based on p-distances (MEGA X) is condensed leaving only branches in which the associated sequences clustered together in over 50 percent of trees (out of 1000 replicates).
