# Supplementary figures and images for: Comparative genomics of the Leukocyte Receptor Complex in carnivores
Source: Front Immunol. 2023 May 10;14:1197687. doi: 10.3389/fimmu.2023.1197687 (PMC10206138; doi:10.3389/fimmu.2023.1197687)

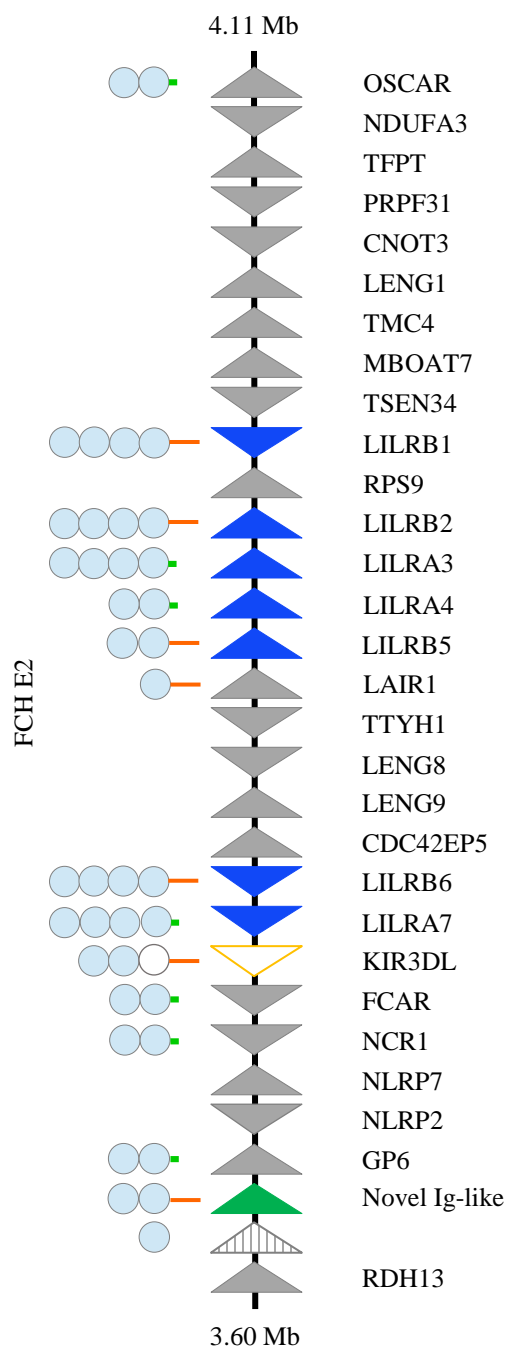

(A)

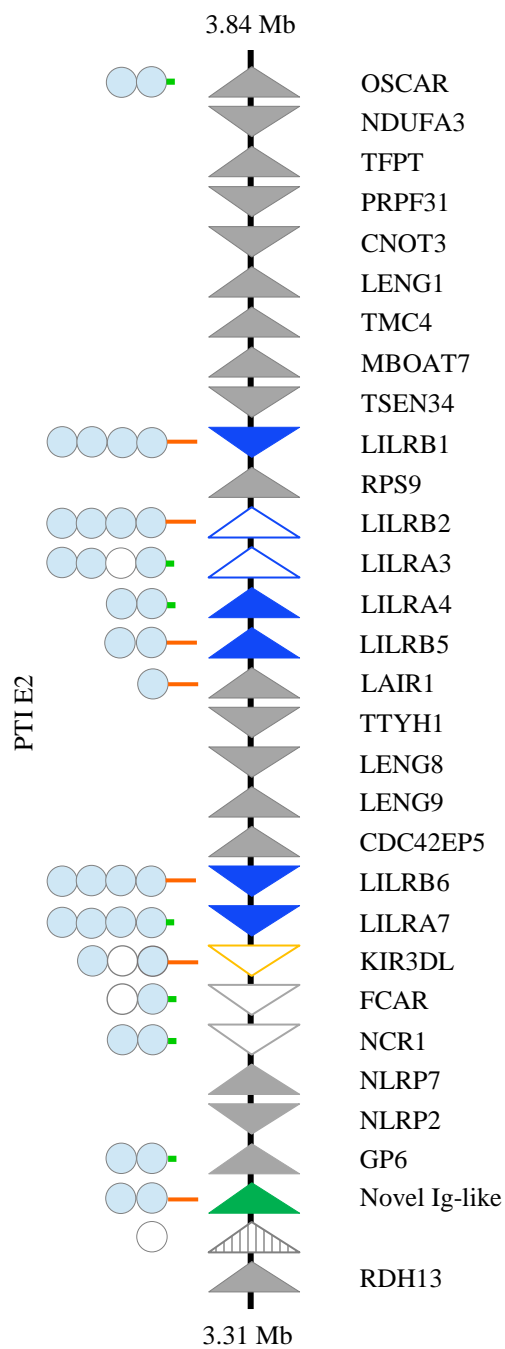

(B)

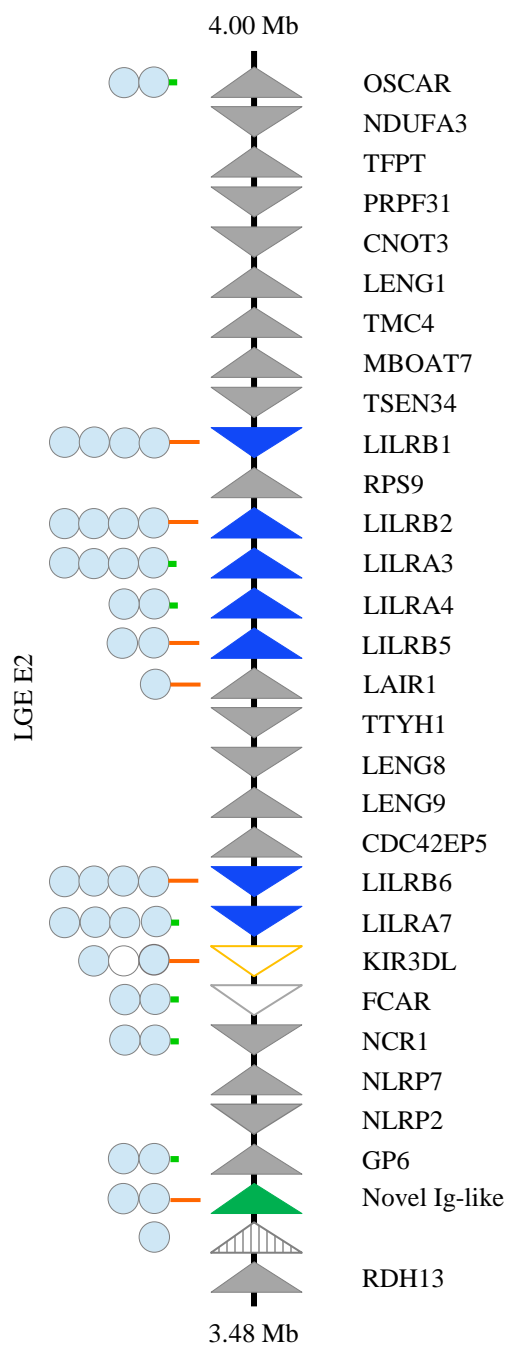

(C)

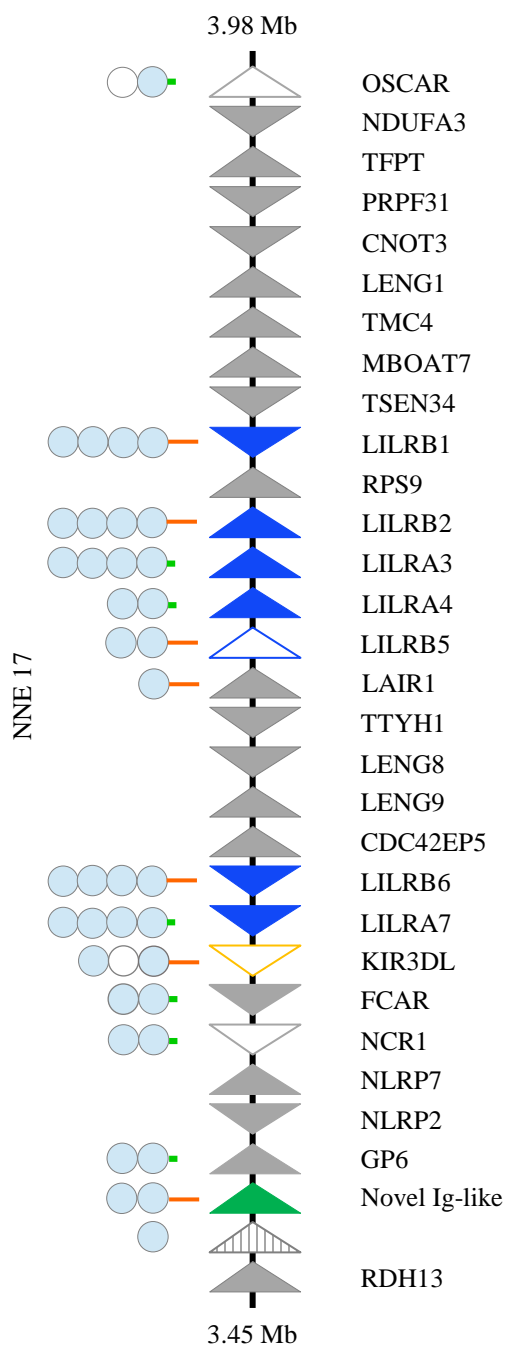

(D)

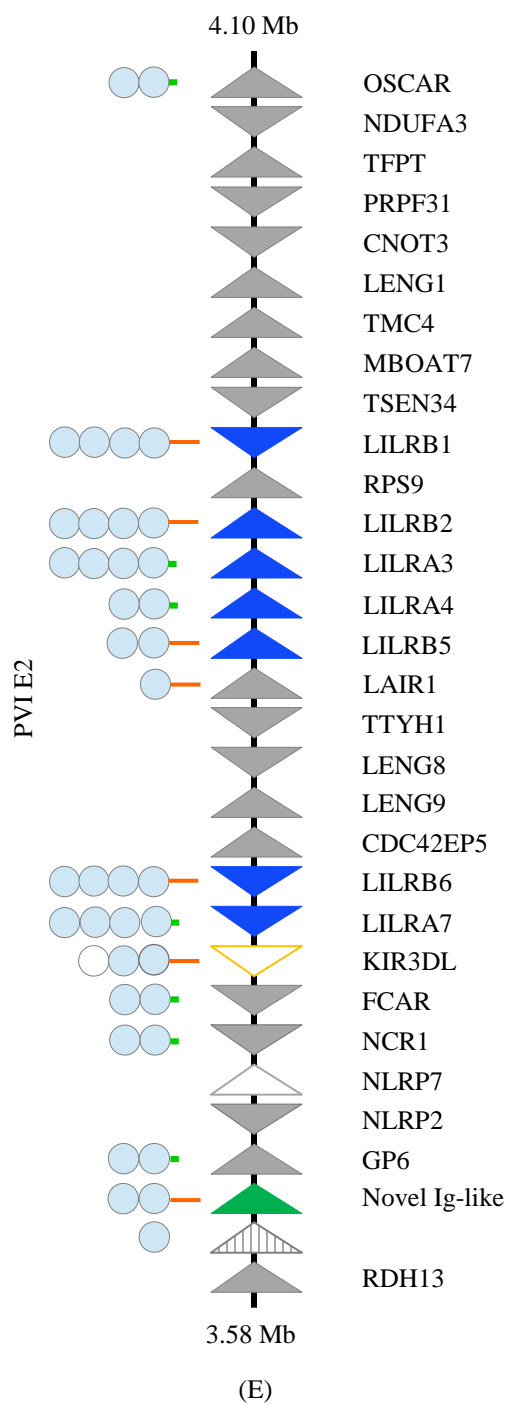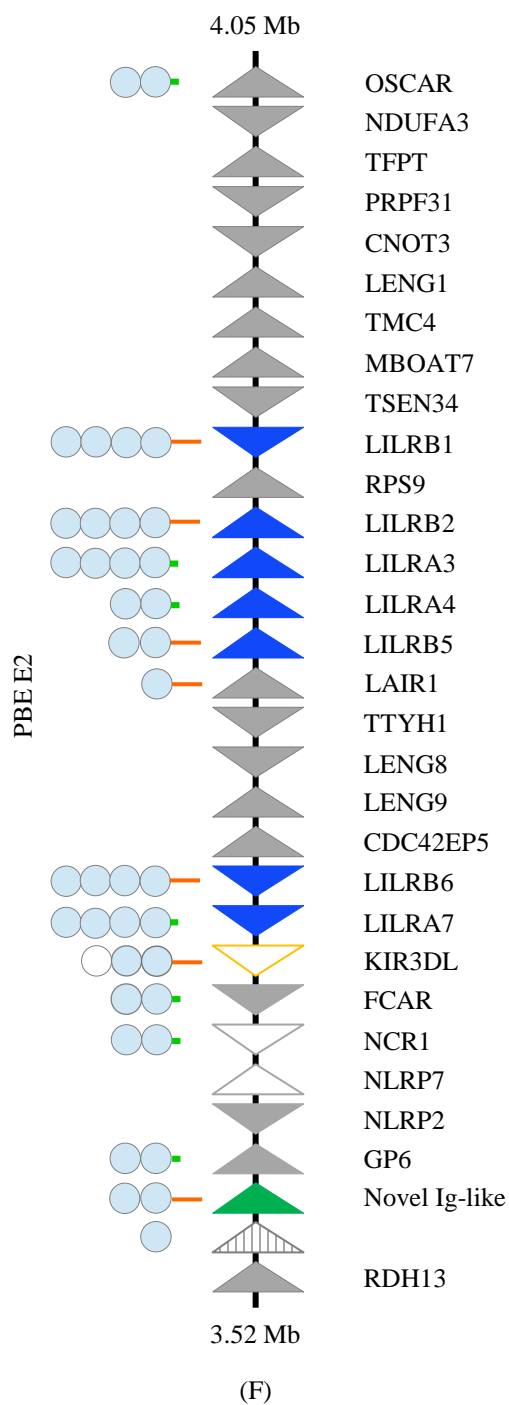

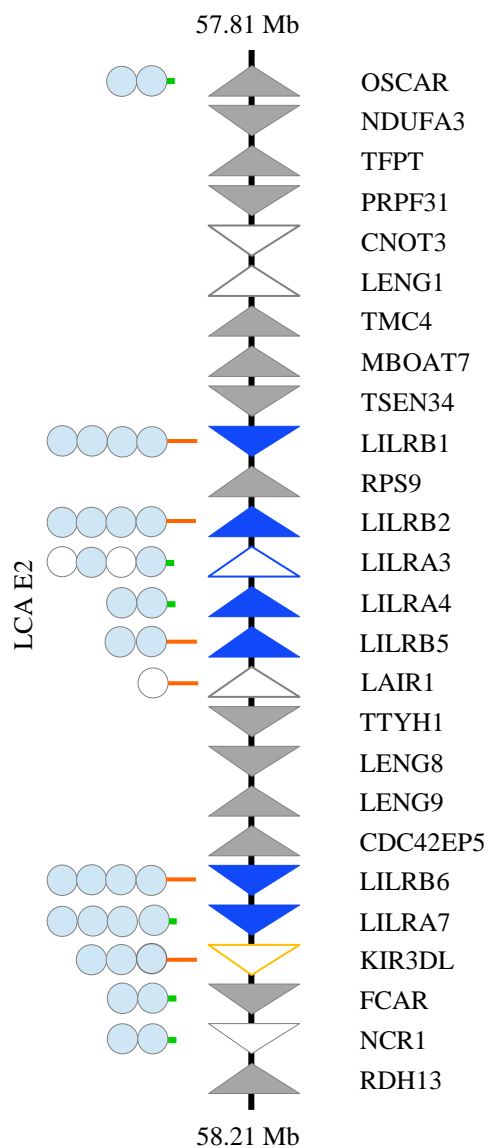

(G)

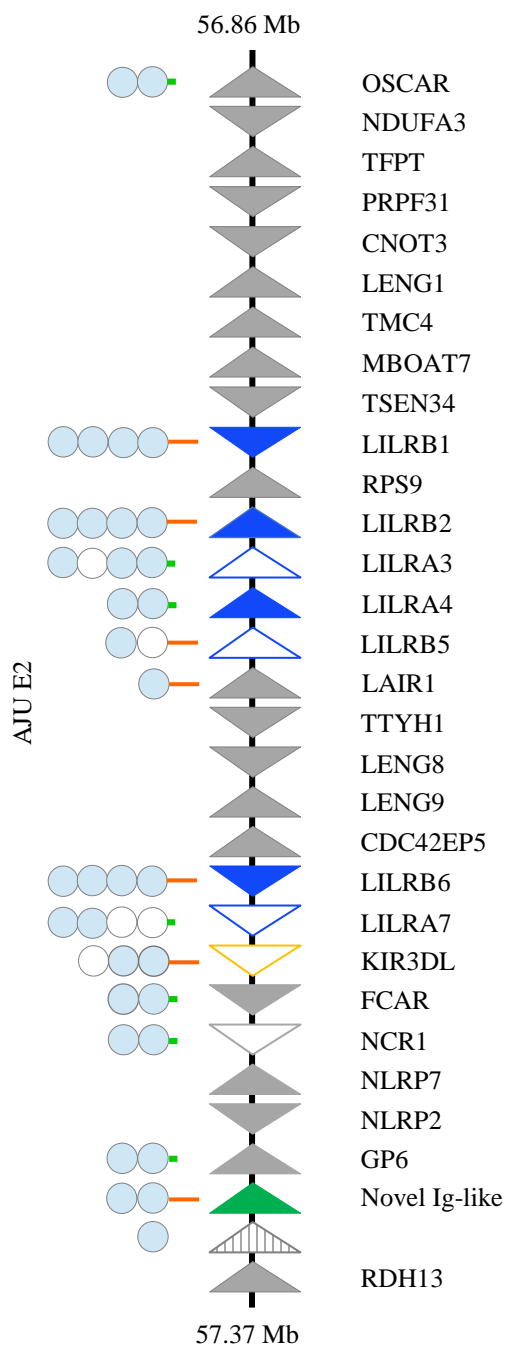

(H)

Supplement: Supplementary file 3 [file Image_1.pdf]

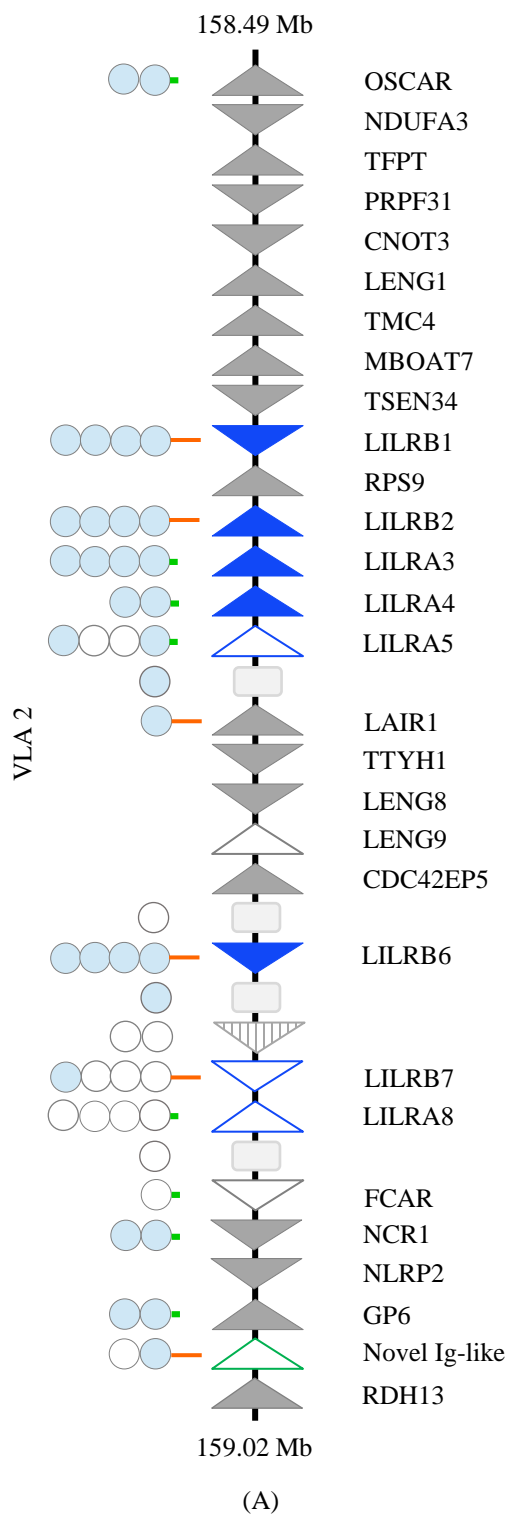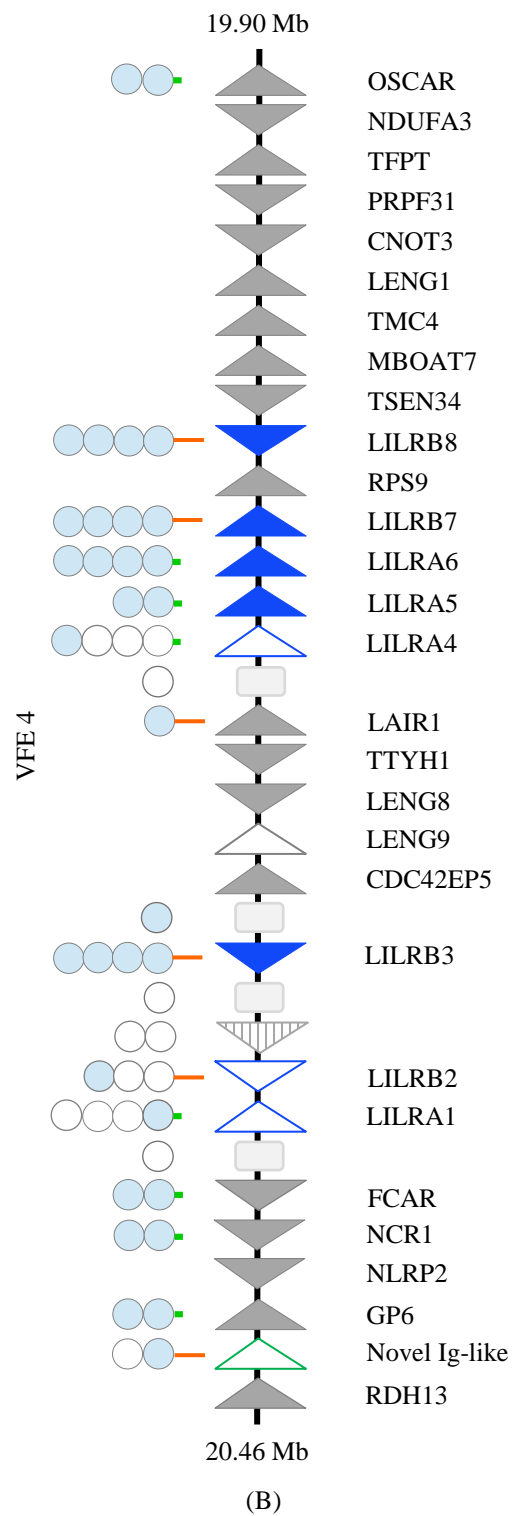

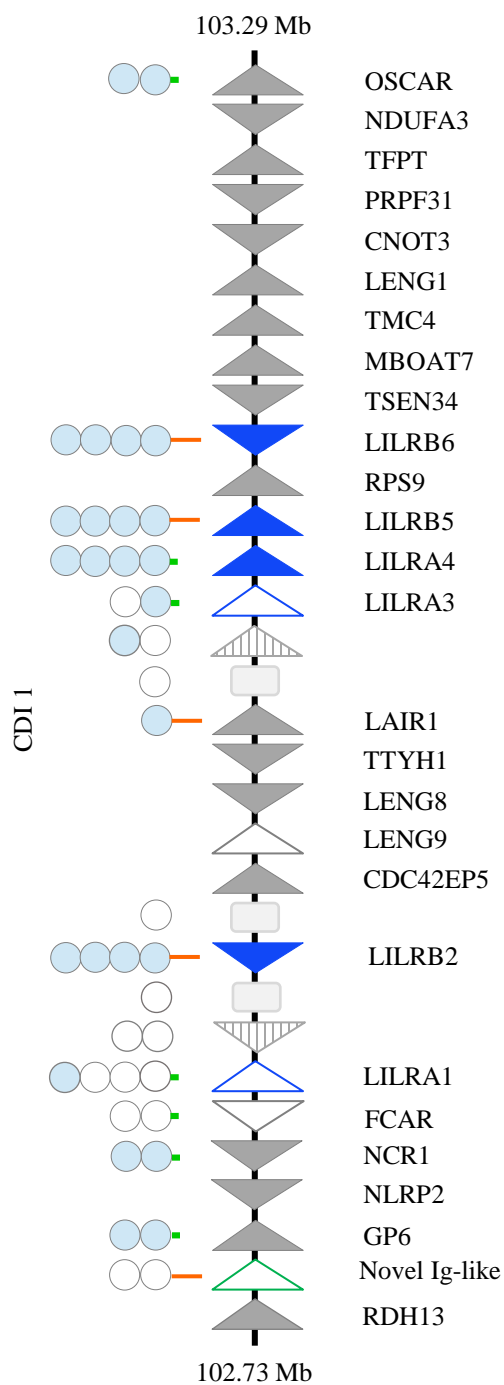

(C)

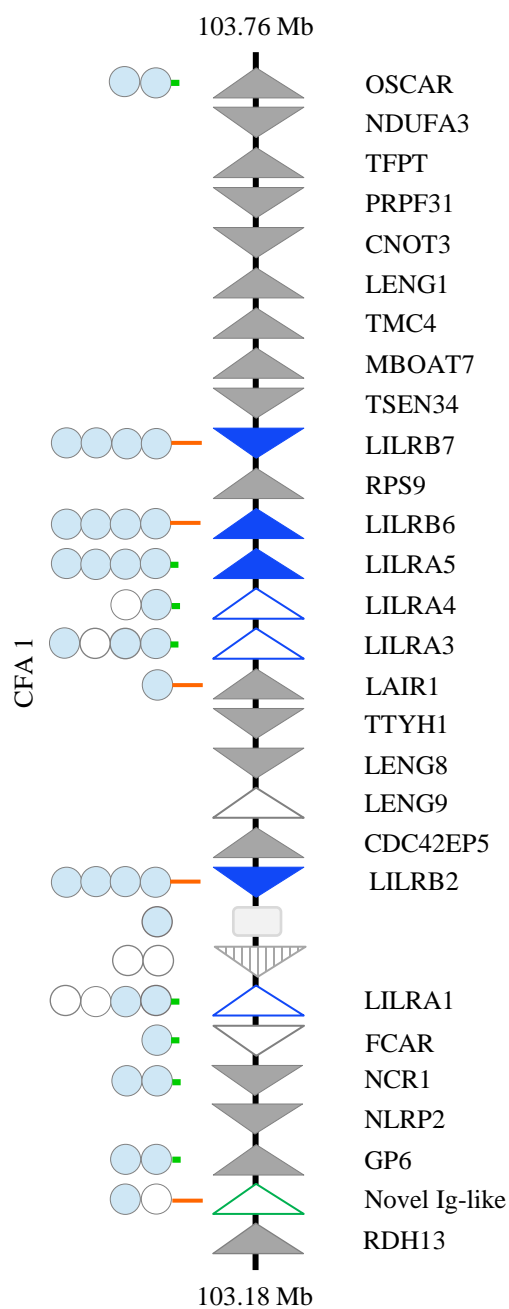

(D)

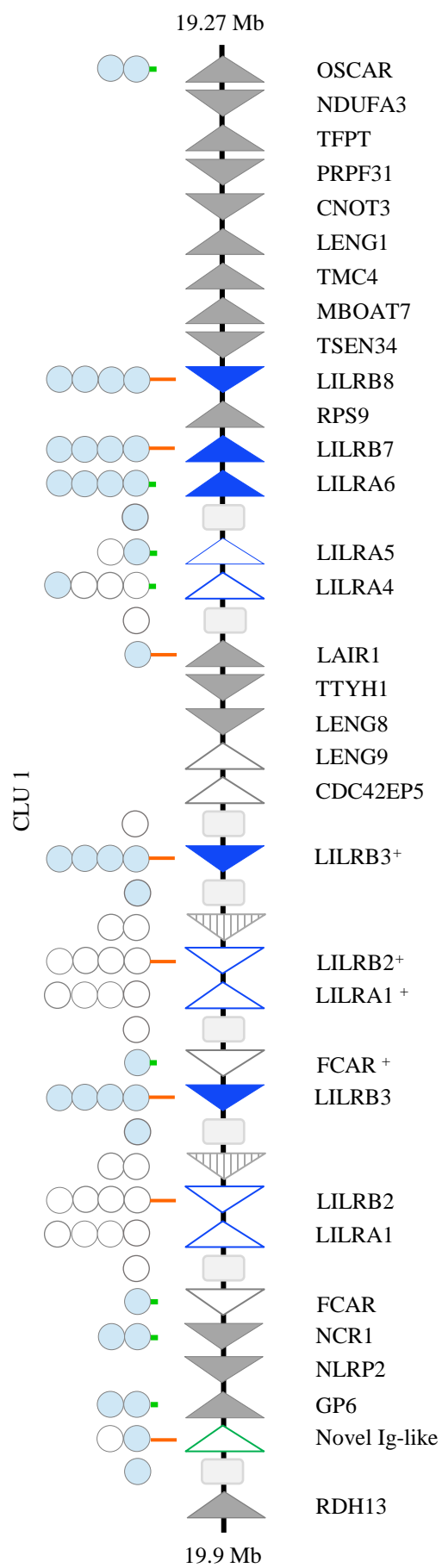

(E)

Supplement: Supplementary file 6 [file Image_4.pdf]

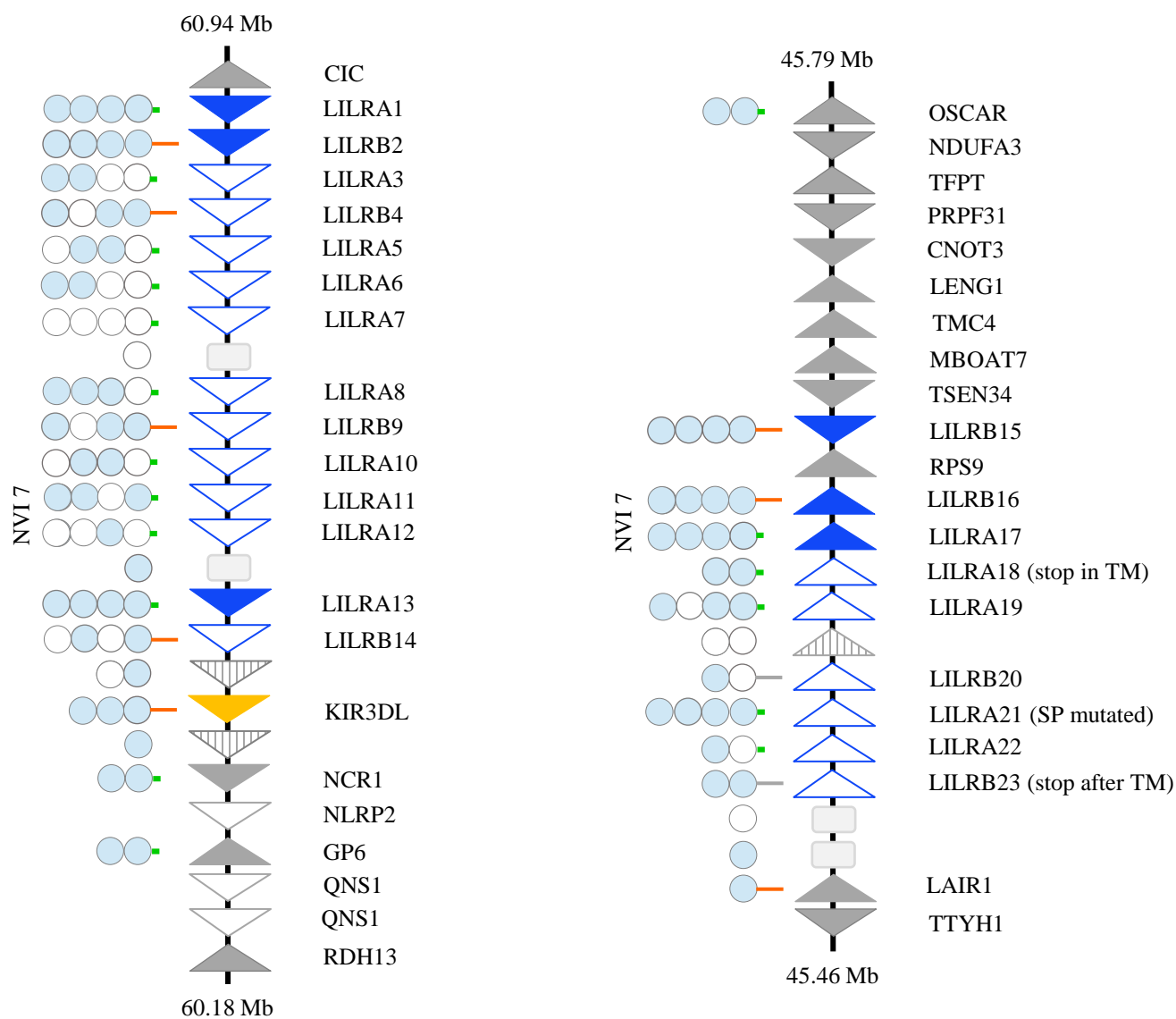

(A)

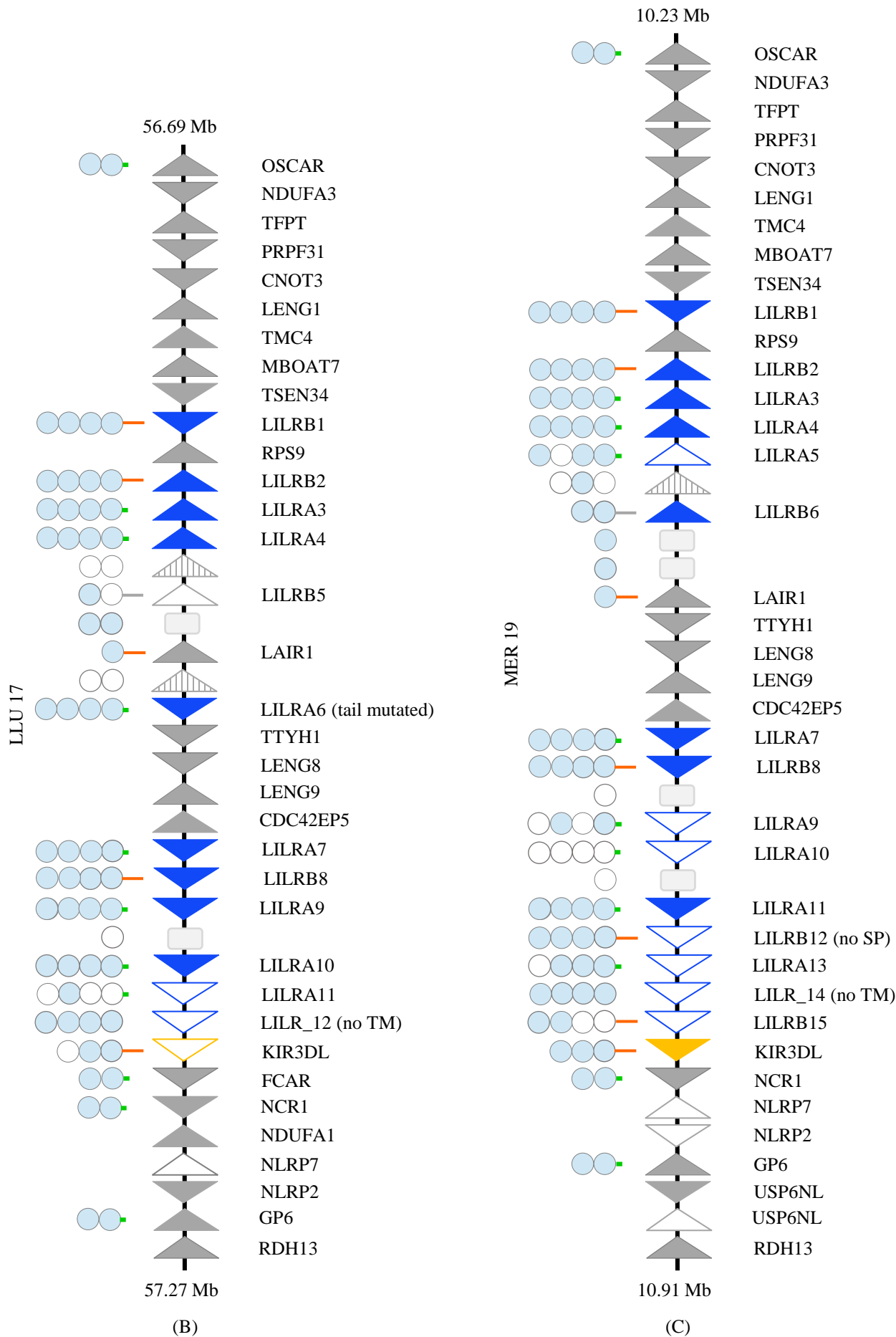

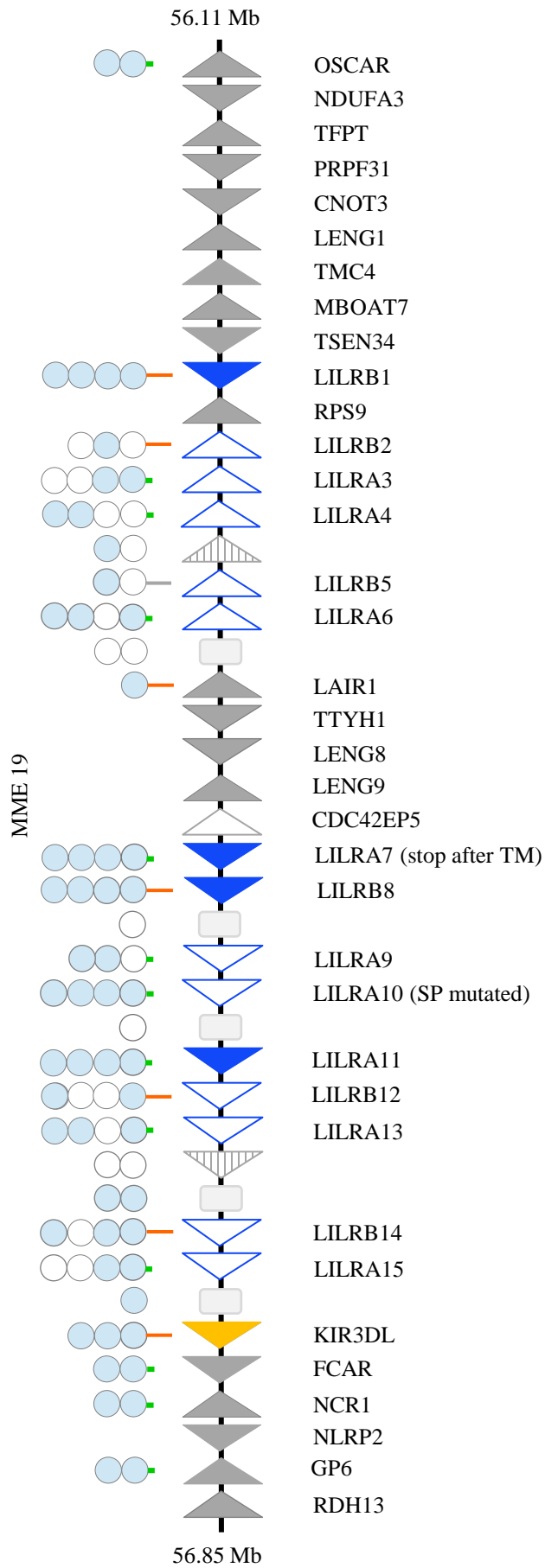

(D)

Supplement: Supplementary file 7 [file Image_5.pdf]

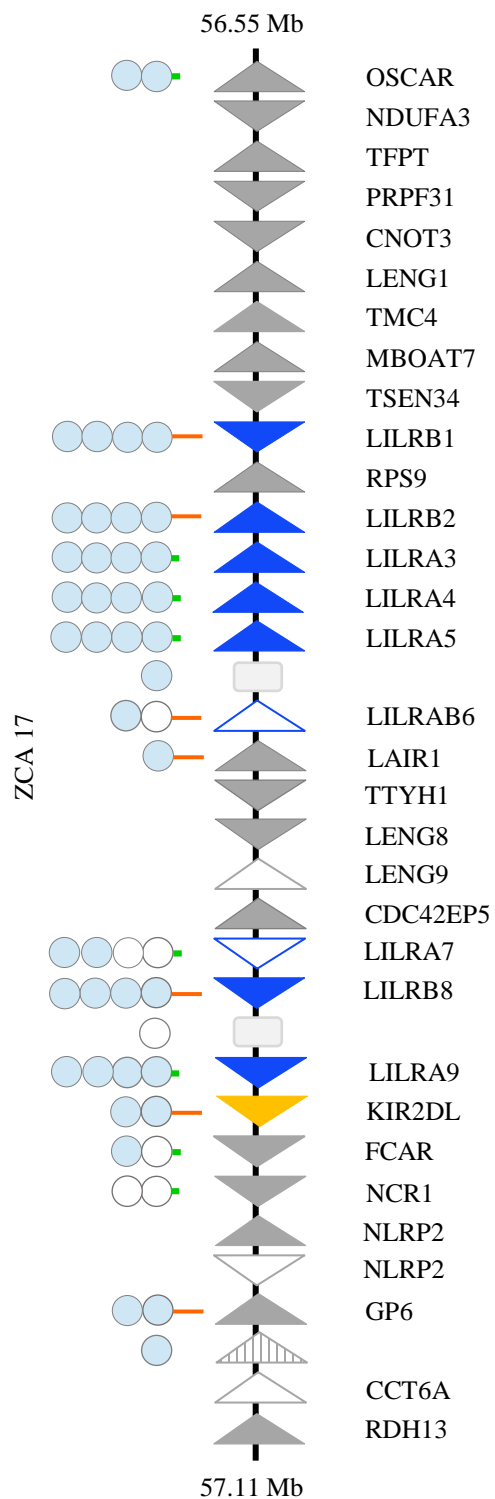

Supplement: Supplementary file 8 [file Image_6.pdf]

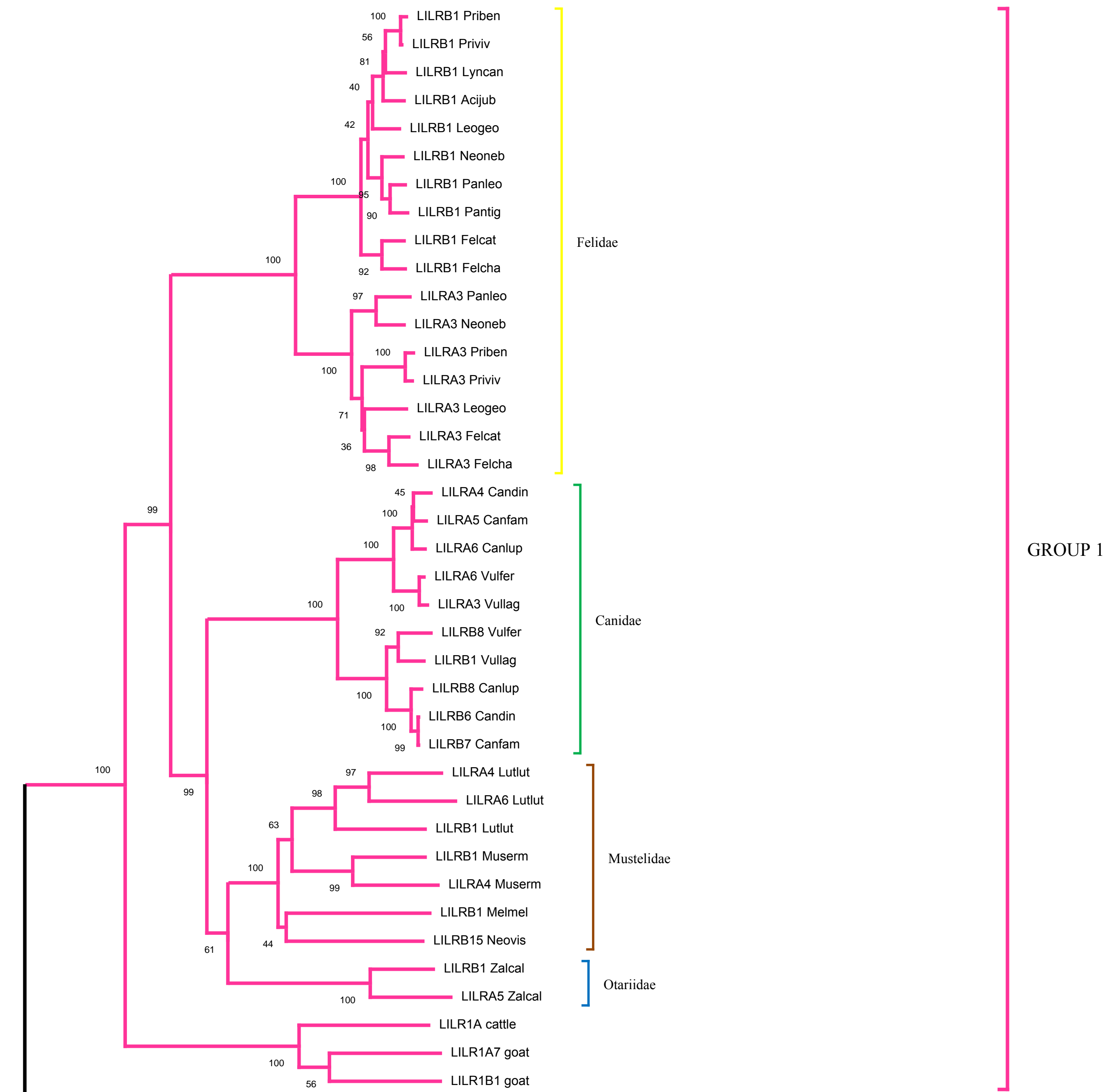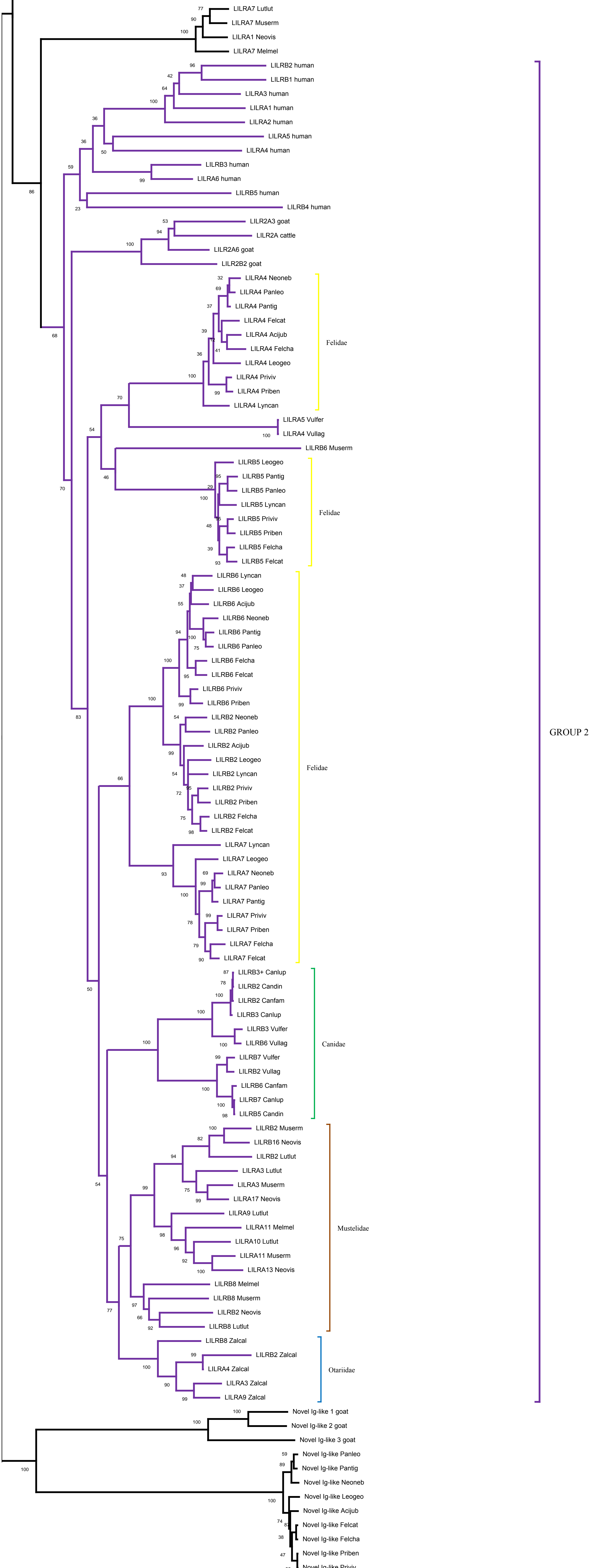

Supplement: Supplementary file 9 [file Image_7.pdf]

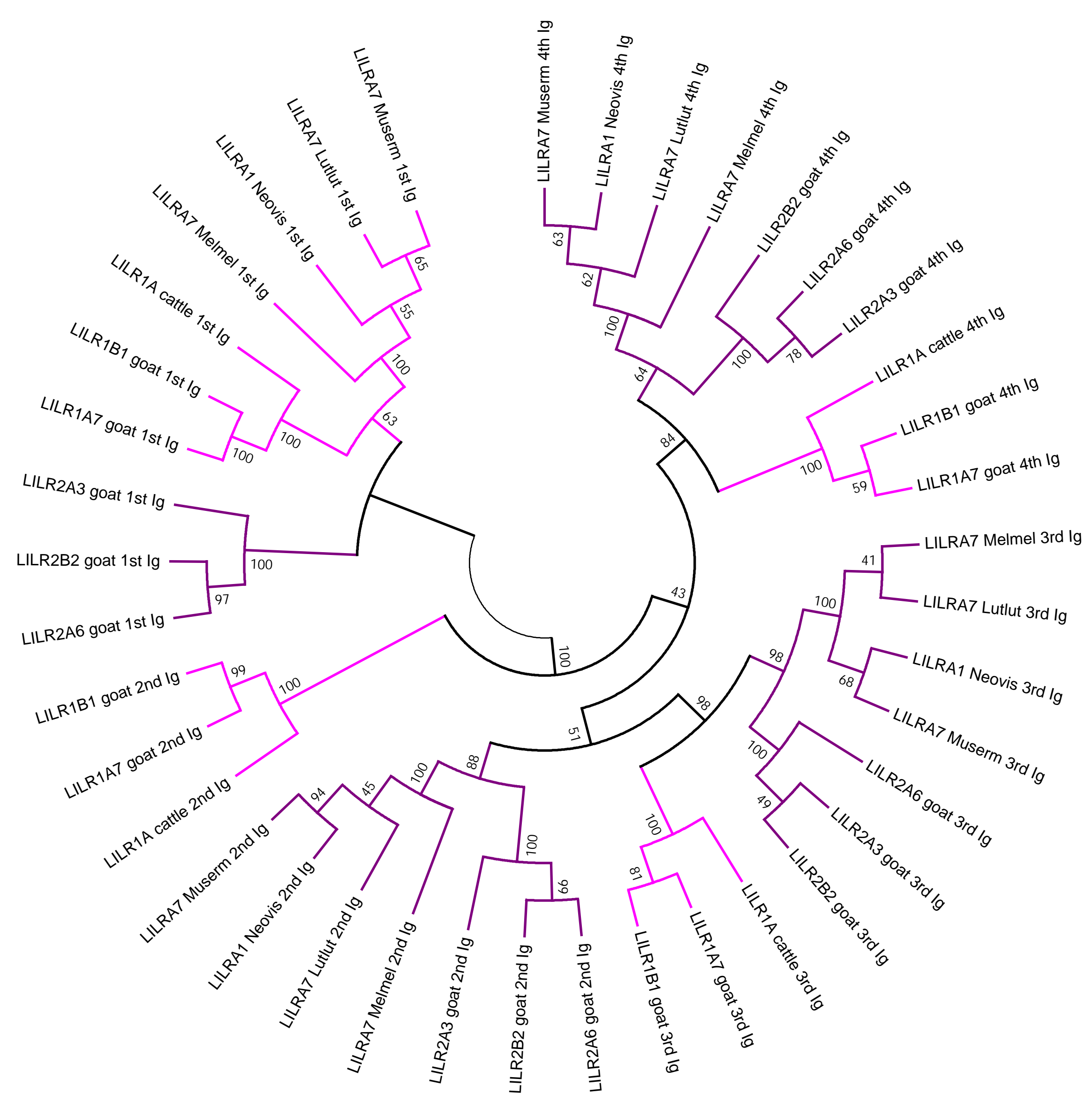

Supplement: Supplementary file 10 [file Image_8.pdf]

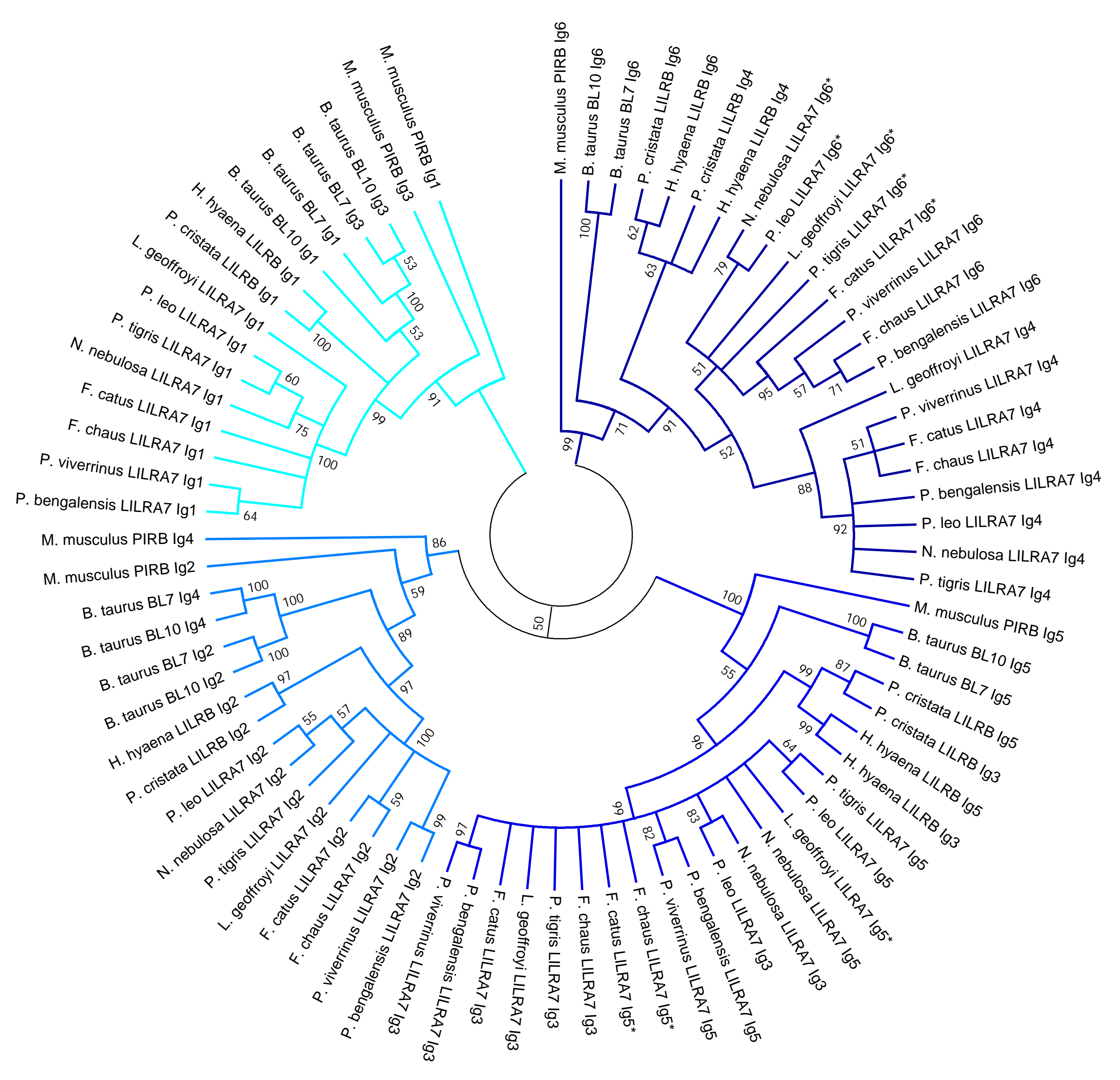

Supplement: Supplementary file 11 [file Image_9.pdf]
