# Supplementary material for: Comparative genomics of the Leukocyte Receptor Complex in carnivores
Source: Front Immunol. 2023 May 10;14:1197687. doi: 10.3389/fimmu.2023.1197687 (PMC10206138; doi:10.3389/fimmu.2023.1197687)
Supplement: Supplementary file 4 [file Image_2.pdf]

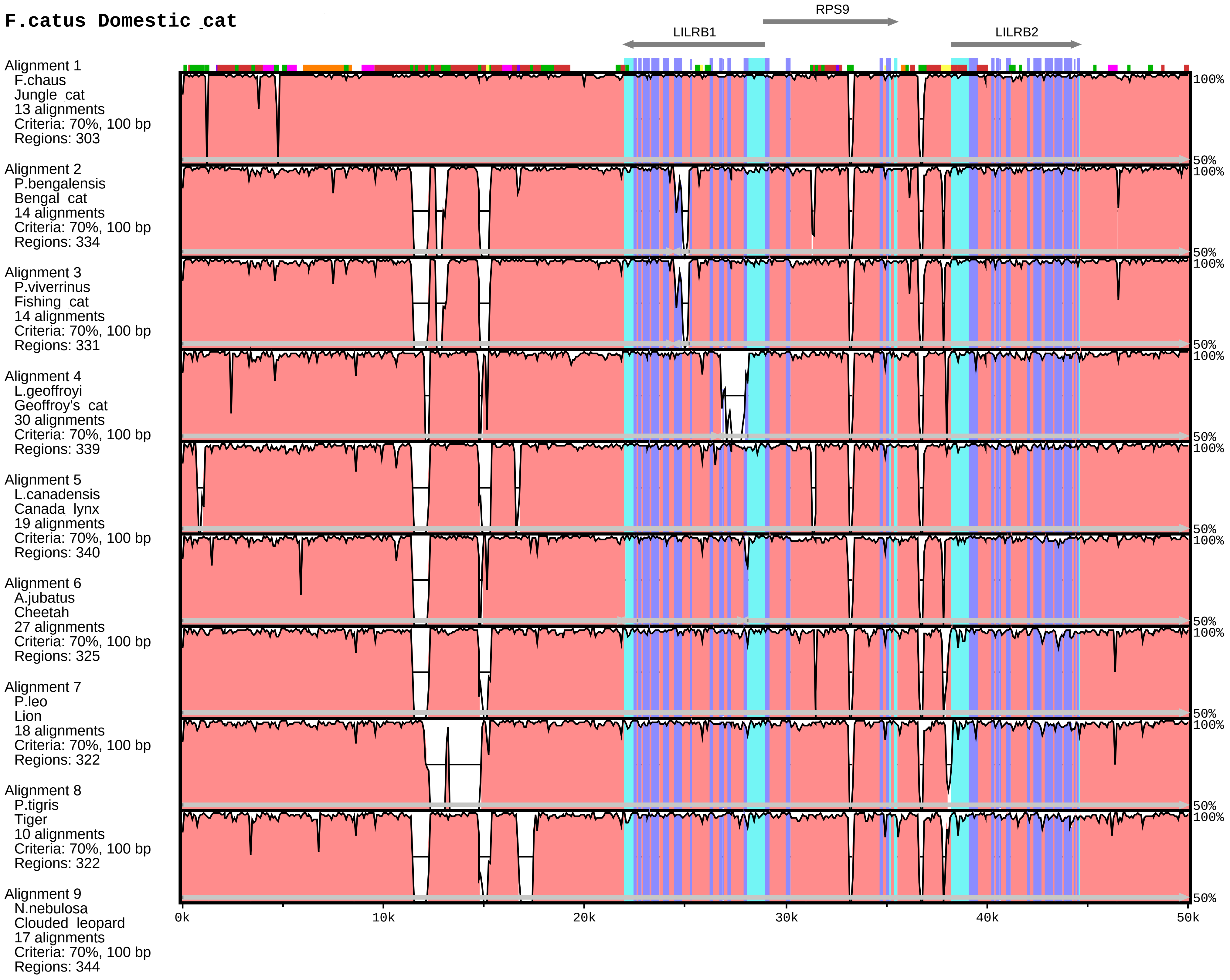
F.catus Domestic cat
RPS9
LILRB1
LILRB2
Alignment 1
F.chaus
Jungle cat
13 alignments
Criteria: 70%, 100 bp
Regions: 303
Alignment 2
P.bengalensis
Bengal cat
14 alignments
Criteria: 70%, 100 bp
Regions: 334
Alignment 3
P.viverrinus
Fishing cat
14 alignments
Criteria: 70%, 100 bp
Regions: 331
Alignment 4
L.geoffroyi
Geoffroy's cat
30 alignments
Criteria: 70%, 100 bp
Regions: 339
Alignment 5
L.canadensis
Canada lynx
19 alignments
Criteria: 70%, 100 bp
Regions: 340
Alignment 6
A.jubatus
Cheetah
27 alignments
Criteria: 70%, 100 bp
Regions: 325
Alignment 7
P.leo
Lion
18 alignments
Criteria: 70%, 100 bp
Regions: 322
Alignment 8
P.tigris
Tiger
10 alignments
Criteria: 70%, 100 bp
Regions: 322
Alignment 9
N.nebulosa
Clouded leopard
17 alignments
Criteria: 70%, 100 bp
Regions: 344
100%
50%
0k
10k
20k
30k
40k
50k

F.catus Domestic_cat

Alignment 1
F.chaus
Jungle cat
13 alignments
Criteria: 70%, 100 bp
Regions: 303

Alignment 2
P.bengalensis
Bengal cat
14 alignments
Criteria: 70%, 100 bp
Regions: 334

Alignment 3
P.viverrinus
Fishing cat
14 alignments
Criteria: 70%, 100 bp
Regions: 331

Alignment 4
L.geoffroyi
Geoffroy's cat
30 alignments
Criteria: 70%, 100 bp
Regions: 339

Alignment 5
L.canadensis
Canada lynx
19 alignments
Criteria: 70%, 100 bp
Regions: 340

Alignment 6
A.jubatus
Cheetah
27 alignments
Criteria: 70%, 100 bp
Regions: 325

Alignment 7
P.leo
Lion
18 alignments
Criteria: 70%, 100 bp
Regions: 322

Alignment 8
P.tigris
Tiger
10 alignments
Criteria: 70%, 100 bp
Regions: 322

Alignment 9
N.nebulosa
Clouded leopard
17 alignments
Criteria: 70%, 100 bp
Regions: 344

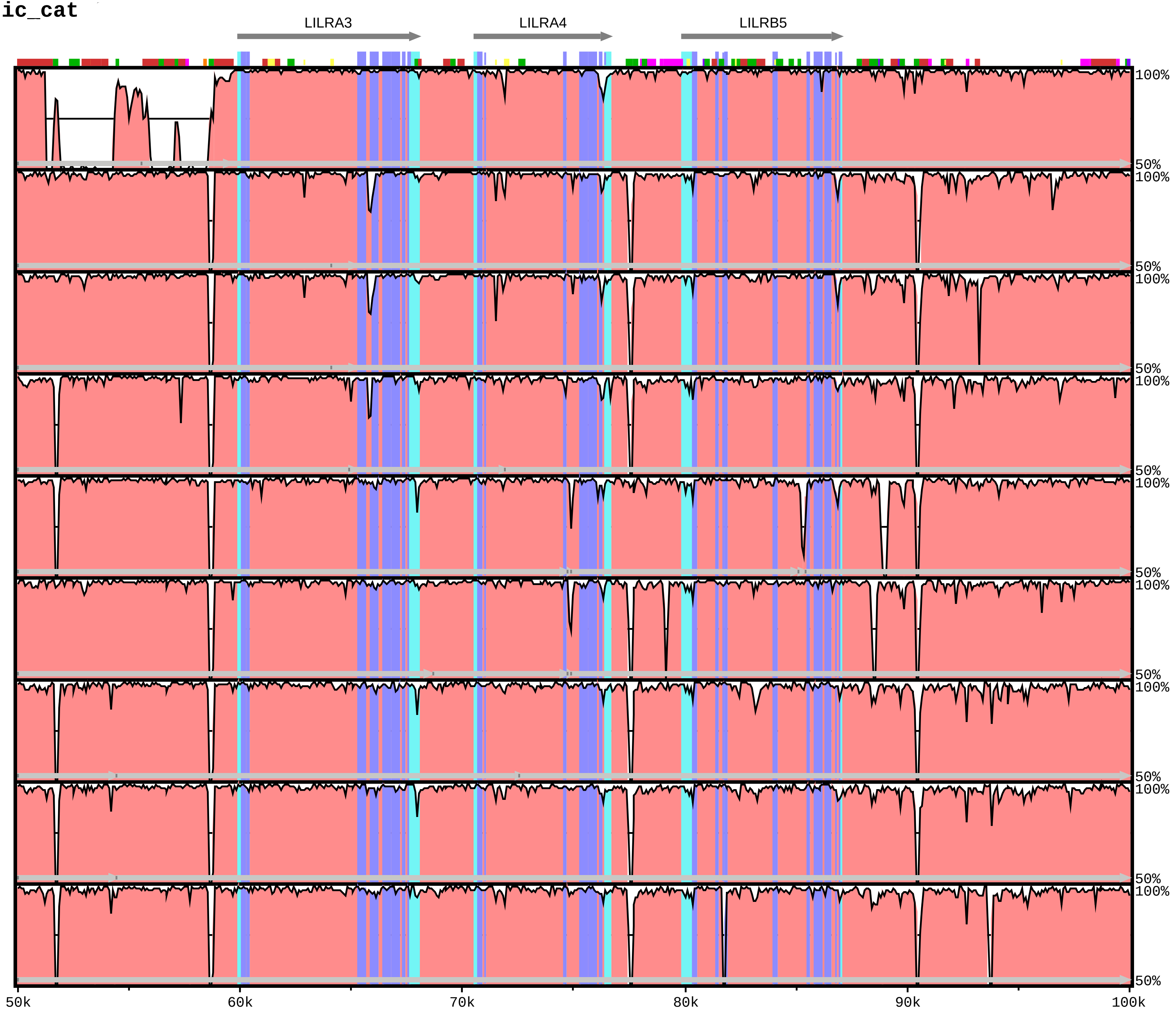

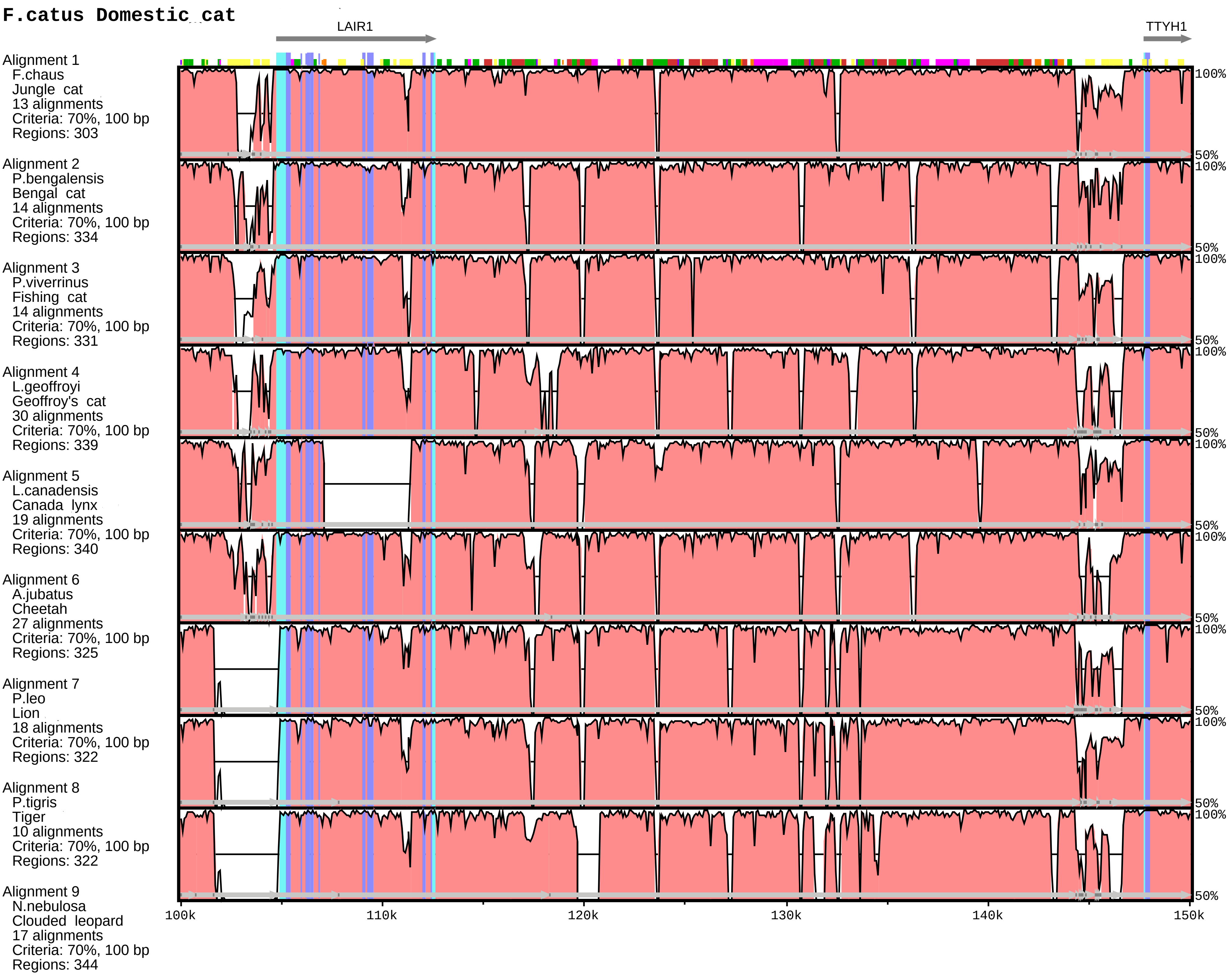
F.catus Domestic cat
LAIR1
TTYH1
Alignment 1
F.chaus
Jungle cat
13 alignments
Criteria: 70%, 100 bp
Regions: 303
Alignment 2
P.bengalensis
Bengal cat
14 alignments
Criteria: 70%, 100 bp
Regions: 334
Alignment 3
P.viverrinus
Fishing cat
14 alignments
Criteria: 70%, 100 bp
Regions: 331
Alignment 4
L.geoffroyi
Geoffroy's cat
30 alignments
Criteria: 70%, 100 bp
Regions: 339
Alignment 5
L.canadensis
Canada lynx
19 alignments
Criteria: 70%, 100 bp
Regions: 340
Alignment 6
A.jubatus
Cheetah
27 alignments
Criteria: 70%, 100 bp
Regions: 325
Alignment 7
P.leo
Lion
18 alignments
Criteria: 70%, 100 bp
Regions: 322
Alignment 8
P.tigris
Tiger
10 alignments
Criteria: 70%, 100 bp
Regions: 322
Alignment 9
N.nebulosa
Clouded leopard
17 alignments
Criteria: 70%, 100 bp
Regions: 344
100%
50%
100k
110k
120k
130k
140k
150k

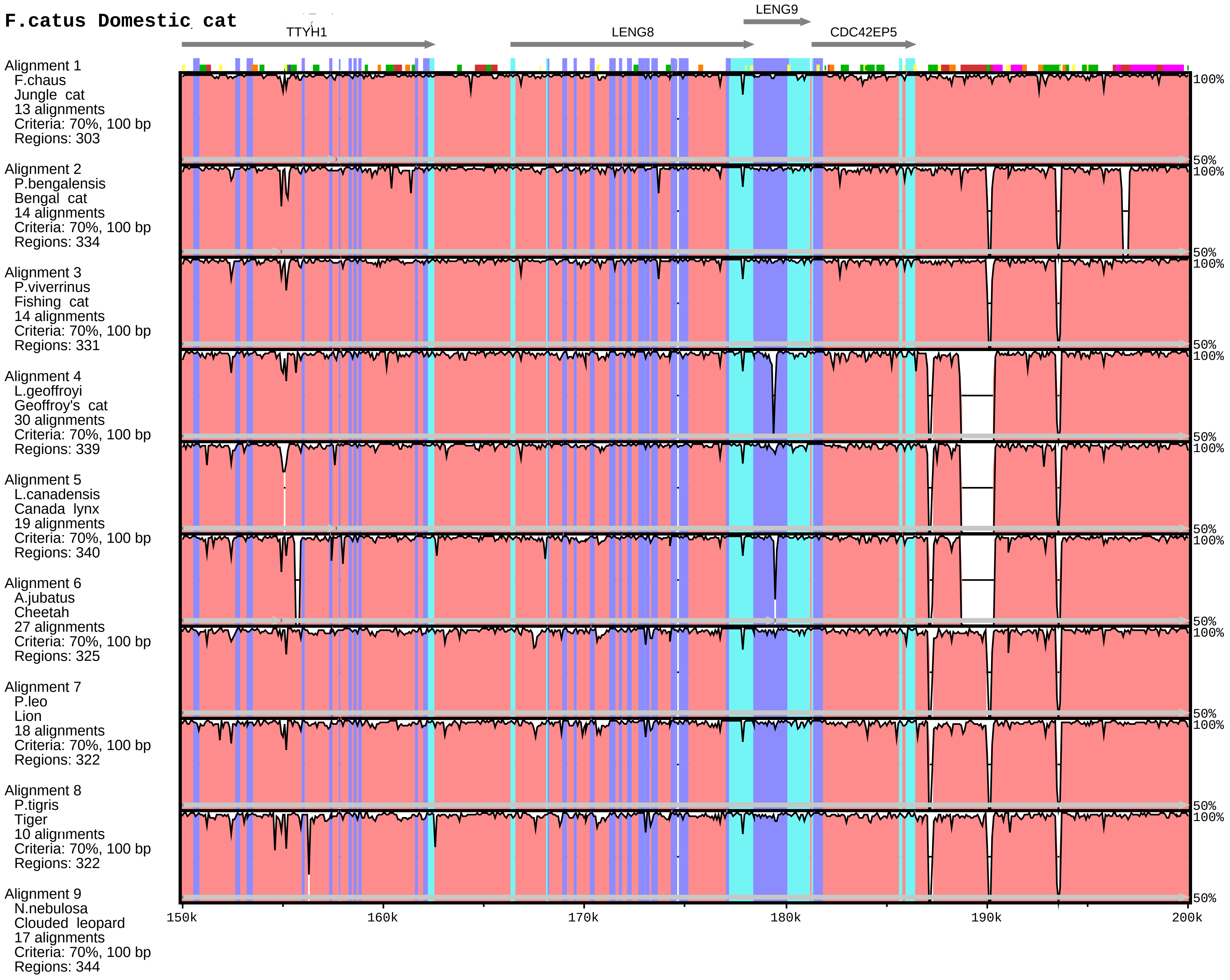
F.catus Domestic cat
TTYH1
LENG8
LENG9
CDC42EP5
Alignment 1
F.chaus
Jungle cat
13 alignments
Criteria: 70%, 100 bp
Regions: 303
Alignment 2
P.bengalensis
Bengal cat
14 alignments
Criteria: 70%, 100 bp
Regions: 334
Alignment 3
P.viverrinus
Fishing cat
14 alignments
Criteria: 70%, 100 bp
Regions: 331
Alignment 4
L.geoffroyi
Geoffroy's cat
30 alignments
Criteria: 70%, 100 bp
Regions: 339
Alignment 5
L.canadensis
Canada lynx
19 alignments
Criteria: 70%, 100 bp
Regions: 340
Alignment 6
A.jubatus
Cheetah
27 alignments
Criteria: 70%, 100 bp
Regions: 325
Alignment 7
P.leo
Lion
18 alignments
Criteria: 70%, 100 bp
Regions: 322
Alignment 8
P.tigris
Tiger
10 alignments
Criteria: 70%, 100 bp
Regions: 322
Alignment 9
N.nebulosa
Clouded leopard
17 alignments
Criteria: 70%, 100 bp
Regions: 344
100%
50%
150k
160k
170k
180k
190k
200k

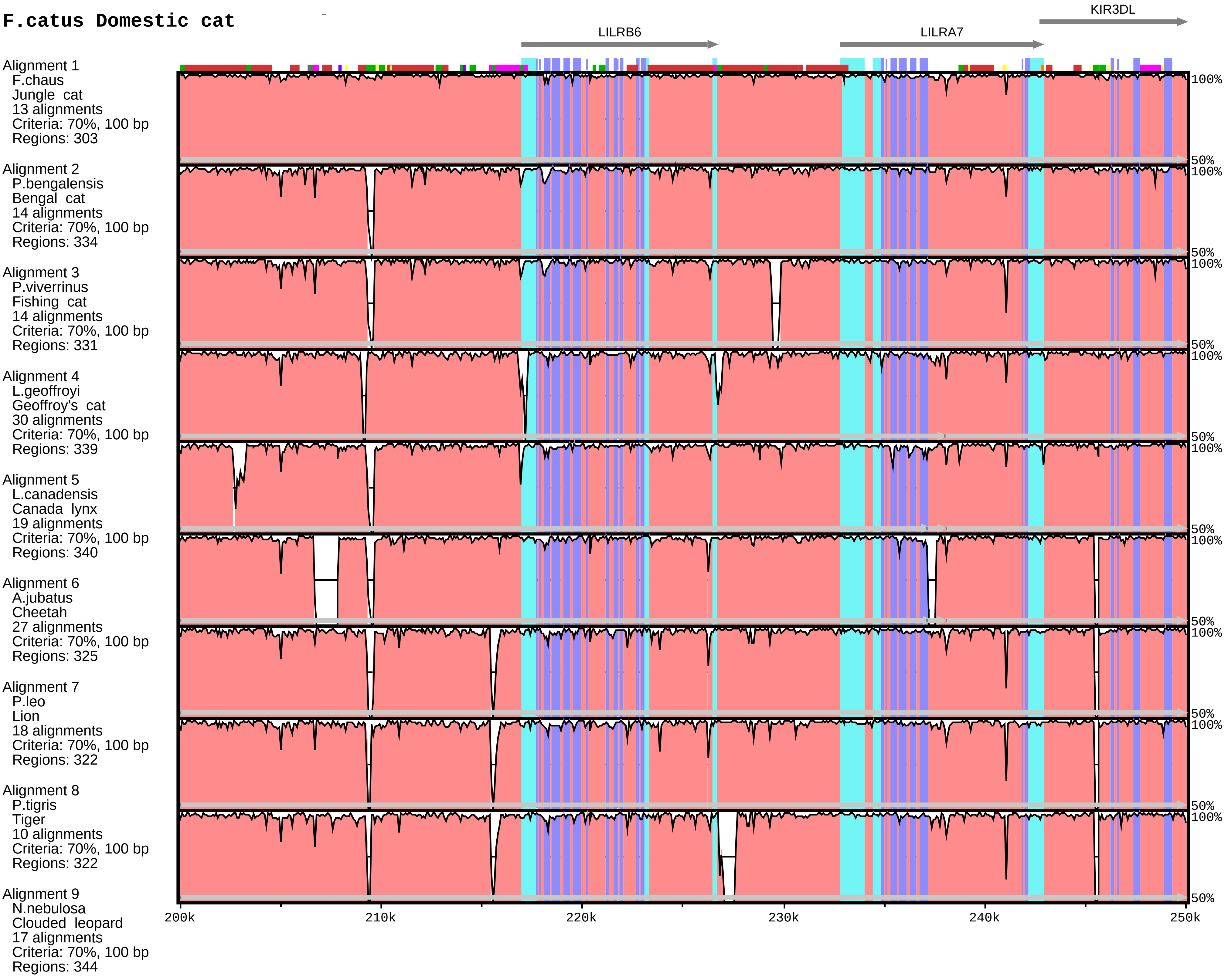
F.catus Domestic cat
LILRB6
LILRA7
KIR3DL
Alignment 1
F.chaus
Jungle cat
13 alignments
Criteria: 70%, 100 bp
Regions: 303
Alignment 2
P.bengalensis
Bengal cat
14 alignments
Criteria: 70%, 100 bp
Regions: 334
Alignment 3
P.viverrinus
Fishing cat
14 alignments
Criteria: 70%, 100 bp
Regions: 331
Alignment 4
L.geoffroyi
Geoffroy's cat
30 alignments
Criteria: 70%, 100 bp
Regions: 339
Alignment 5
L.canadensis
Canada lynx
19 alignments
Criteria: 70%, 100 bp
Regions: 340
Alignment 6
A.jubatus
Cheetah
27 alignments
Criteria: 70%, 100 bp
Regions: 325
Alignment 7
P.leo
Lion
18 alignments
Criteria: 70%, 100 bp
Regions: 322
Alignment 8
P.tigris
Tiger
10 alignments
Criteria: 70%, 100 bp
Regions: 322
Alignment 9
N.nebulosa
Clouded leopard
17 alignments
Criteria: 70%, 100 bp
Regions: 344
100%
50%
200k
210k
220k
230k
240k
250k

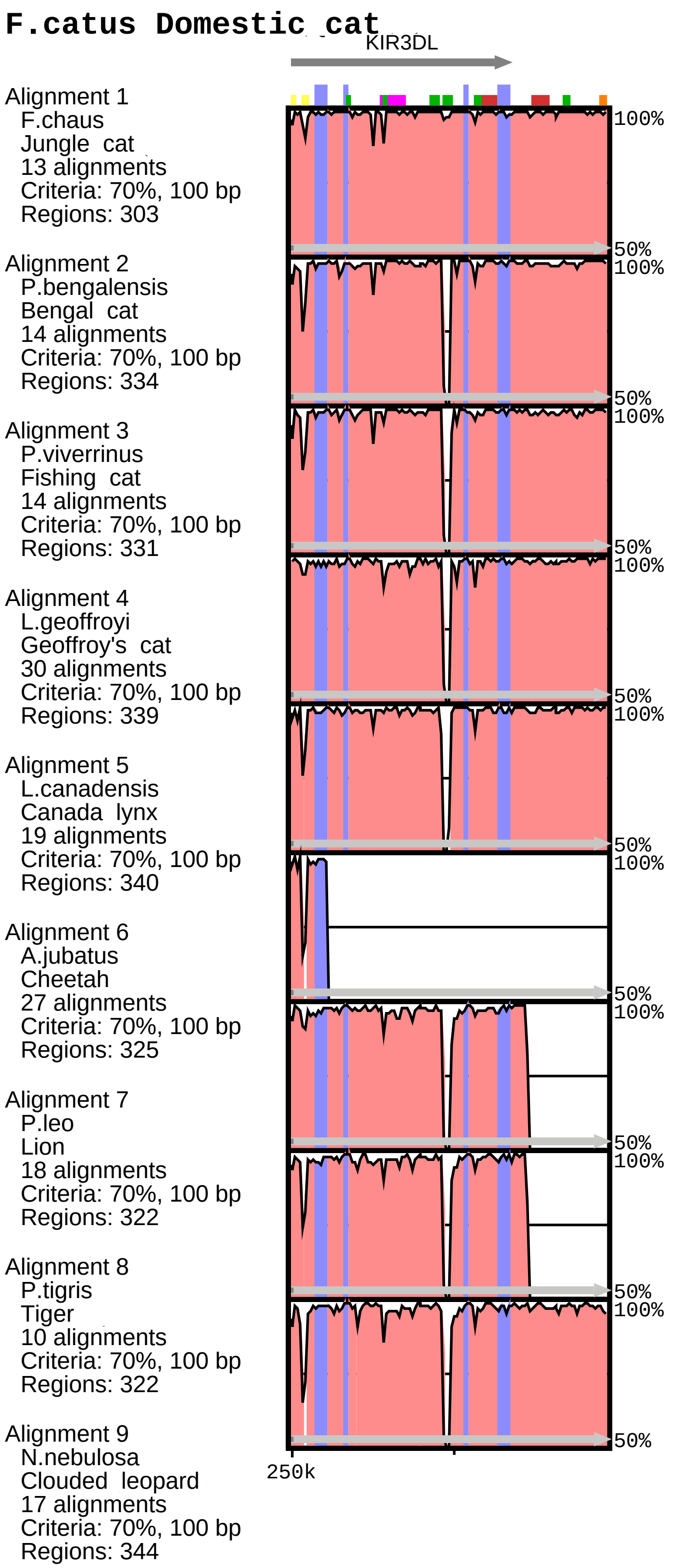
F.catus Domestic cat
KIR3DL
Alignment 1
F.chaus
Jungle cat
13 alignments
Criteria: 70%, 100 bp
Regions: 303
Alignment 2
P.bengalensis
Bengal cat
14 alignments
Criteria: 70%, 100 bp
Regions: 334
Alignment 3
P.viverrinus
Fishing cat
14 alignments
Criteria: 70%, 100 bp
Regions: 331
Alignment 4
L.geoffroyi
Geoffroy's cat
30 alignments
Criteria: 70%, 100 bp
Regions: 339
Alignment 5
L.canadensis
Canada lynx
19 alignments
Criteria: 70%, 100 bp
Regions: 340
Alignment 6
A.jubatus
Cheetah
27 alignments
Criteria: 70%, 100 bp
Regions: 325
Alignment 7
P.leo
Lion
18 alignments
Criteria: 70%, 100 bp
Regions: 322
Alignment 8
P.tigris
Tiger
10 alignments
Criteria: 70%, 100 bp
Regions: 322
Alignment 9
N.nebulosa
Clouded leopard
17 alignments
Criteria: 70%, 100 bp
Regions: 344
100%
50%
250k
