# Supplementary material for: Comparative genomics of the Leukocyte Receptor Complex in carnivores
Source: Front Immunol. 2023 May 10;14:1197687. doi: 10.3389/fimmu.2023.1197687 (PMC10206138; doi:10.3389/fimmu.2023.1197687)
Supplement: Supplementary file 5 [file Image_3.pdf]

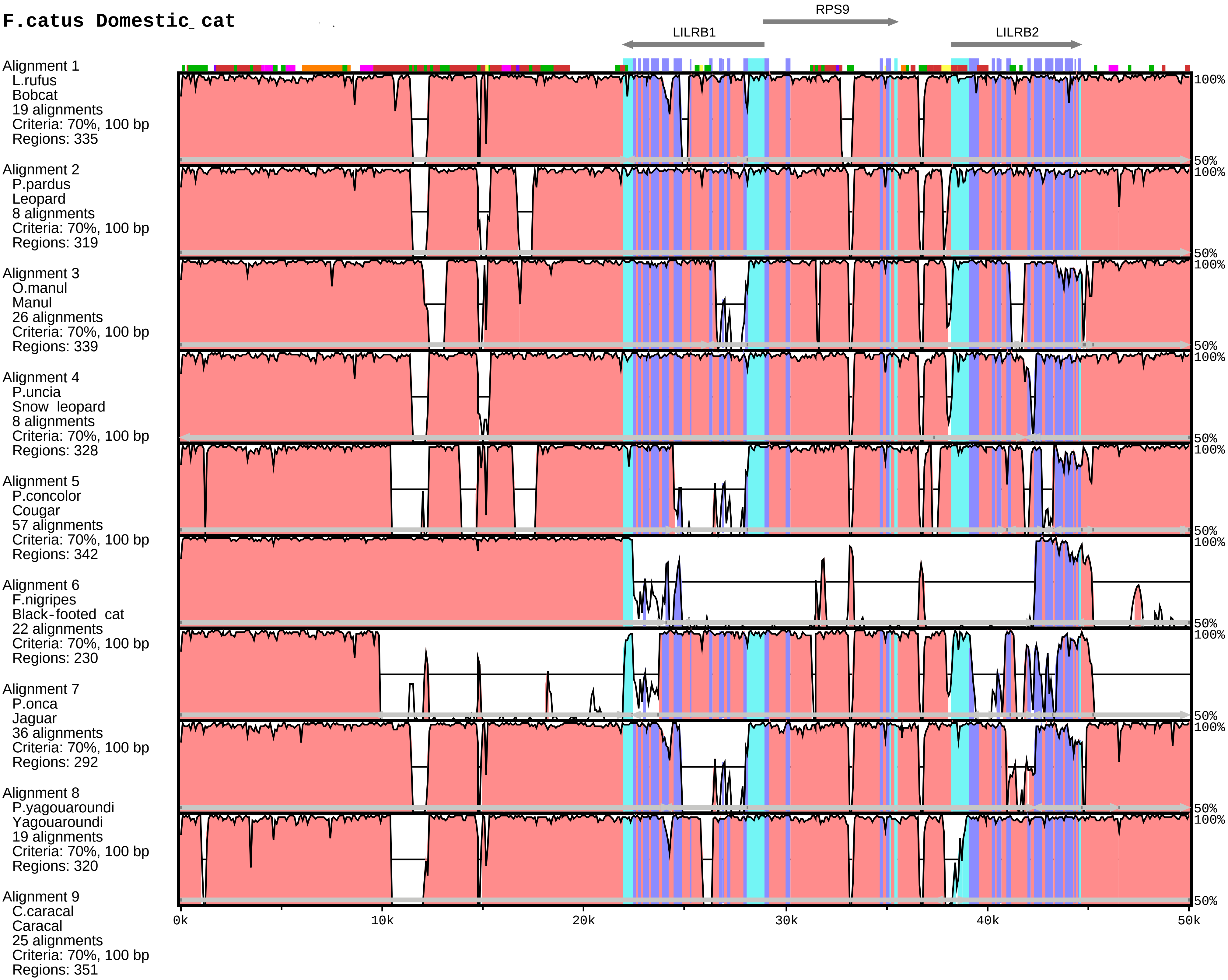
F.catus Domestic_cat
RPS9
LILRB1
LILRB2
Alignment 1
L.rufus
Bobcat
19 alignments
Criteria: 70%, 100 bp
Regions: 335
Alignment 2
P.pardus
Leopard
8 alignments
Criteria: 70%, 100 bp
Regions: 319
Alignment 3
O.manul
Manul
26 alignments
Criteria: 70%, 100 bp
Regions: 339
Alignment 4
P.uncia
Snow leopard
8 alignments
Criteria: 70%, 100 bp
Regions: 328
Alignment 5
P.concolor
Cougar
57 alignments
Criteria: 70%, 100 bp
Regions: 342
Alignment 6
F.nigripes
Black-footed cat
22 alignments
Criteria: 70%, 100 bp
Regions: 230
Alignment 7
P.onca
Jaguar
36 alignments
Criteria: 70%, 100 bp
Regions: 292
Alignment 8
P.yagouaroundi
Yagouaroundi
19 alignments
Criteria: 70%, 100 bp
Regions: 320
Alignment 9
C.caracal
Caracal
25 alignments
Criteria: 70%, 100 bp
Regions: 351
100%
50%
0k
10k
20k
30k
40k
50k

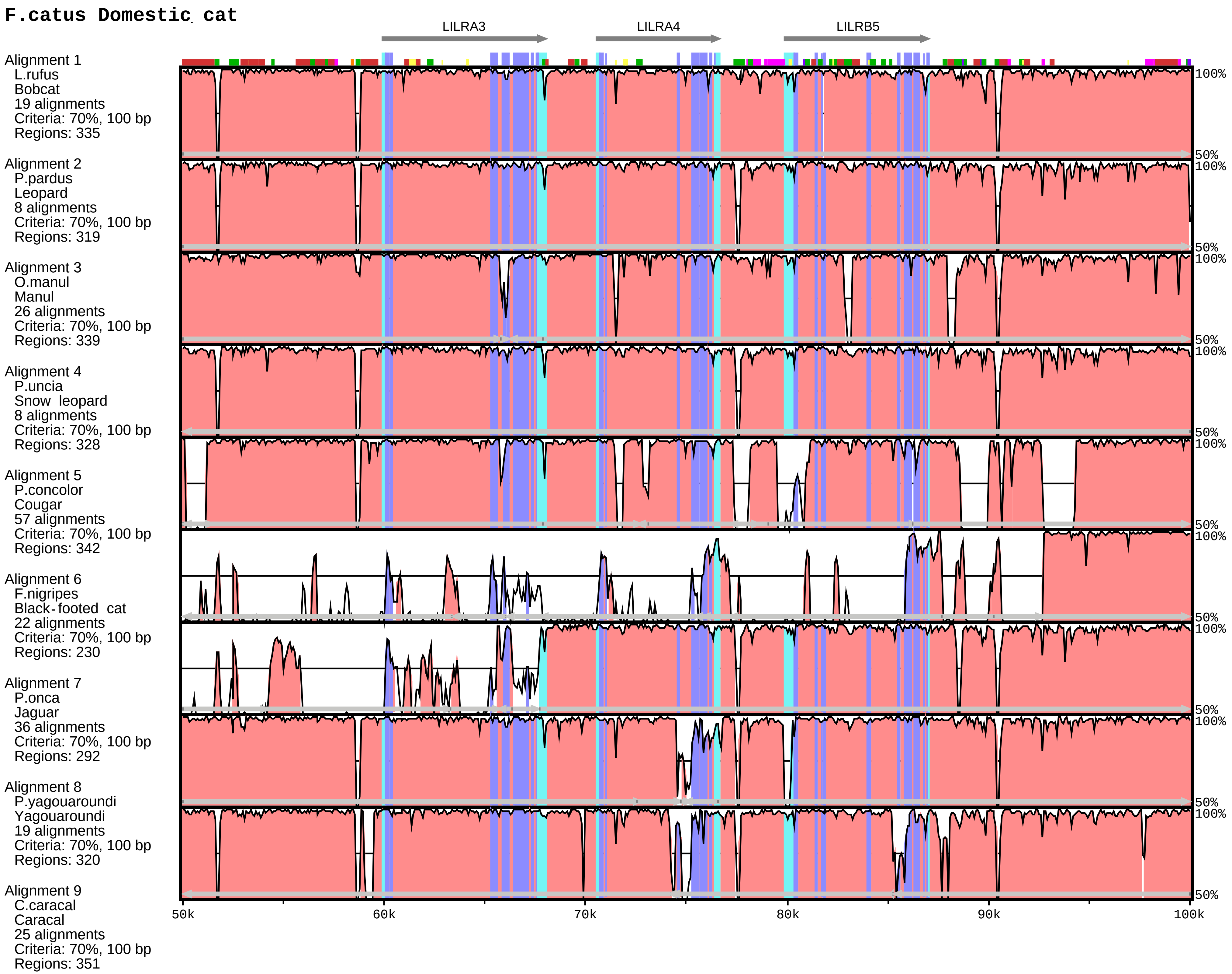
F.catus Domestic cat
LILRA3
LILRA4
LILRB5
Alignment 1
L.rufus
Bobcat
19 alignments
Criteria: 70%, 100 bp
Regions: 335
Alignment 2
P.pardus
Leopard
8 alignments
Criteria: 70%, 100 bp
Regions: 319
Alignment 3
O.manul
Manul
26 alignments
Criteria: 70%, 100 bp
Regions: 339
Alignment 4
P.uncia
Snow leopard
8 alignments
Criteria: 70%, 100 bp
Regions: 328
Alignment 5
P.concolor
Cougar
57 alignments
Criteria: 70%, 100 bp
Regions: 342
Alignment 6
F.nigripes
Black-footed cat
22 alignments
Criteria: 70%, 100 bp
Regions: 230
Alignment 7
P.onca
Jaguar
36 alignments
Criteria: 70%, 100 bp
Regions: 292
Alignment 8
P.yagouaroundi
Yagouaroundi
19 alignments
Criteria: 70%, 100 bp
Regions: 320
Alignment 9
C.caracal
Caracal
25 alignments
Criteria: 70%, 100 bp
Regions: 351
100%
50%
50k
60k
70k
80k
90k
100k

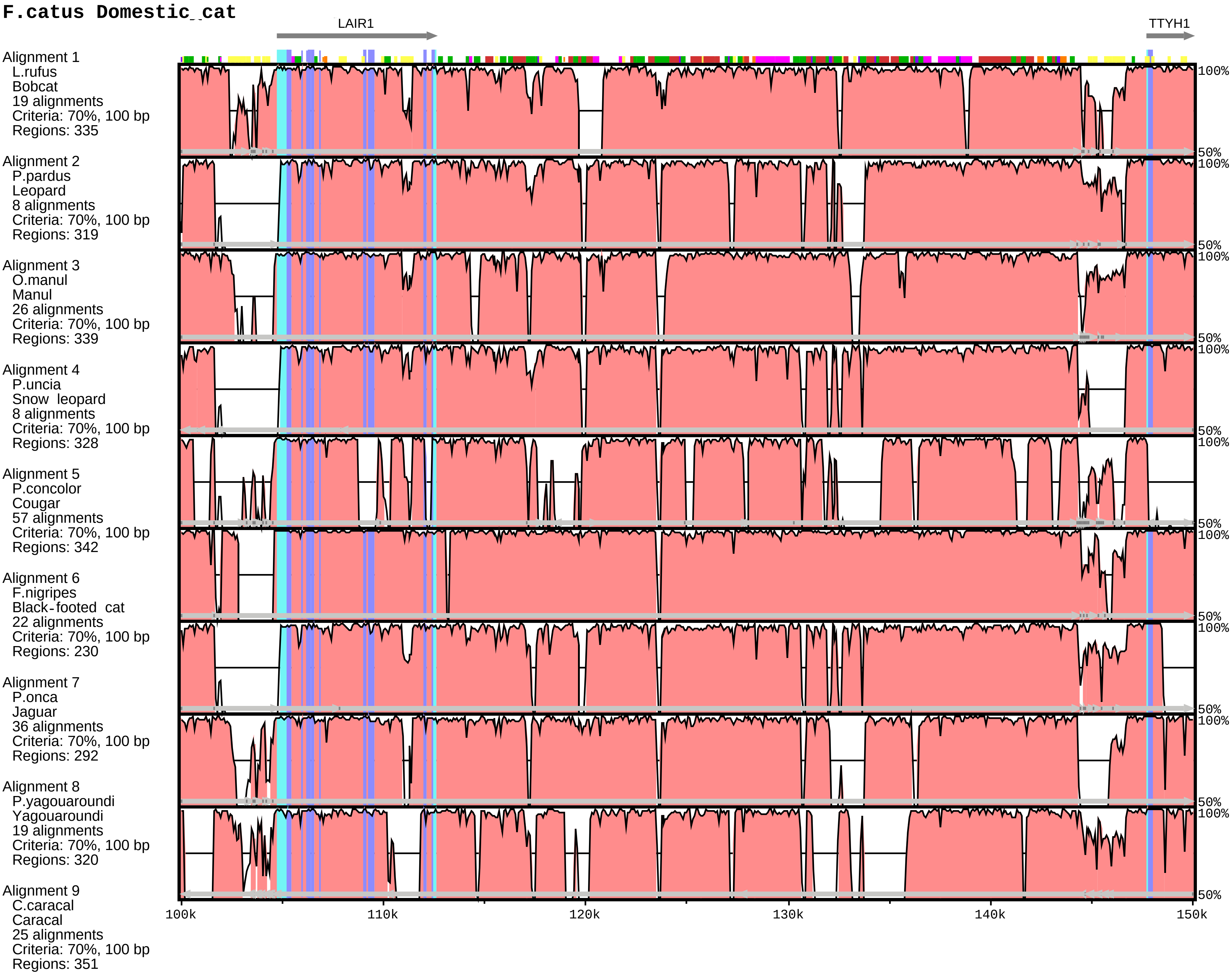
F.catus Domestic_cat
LAIR1
TTYH1
Alignment 1
L.rufus
Bobcat
19 alignments
Criteria: 70%, 100 bp
Regions: 335
Alignment 2
P.pardus
Leopard
8 alignments
Criteria: 70%, 100 bp
Regions: 319
Alignment 3
O.manul
Manul
26 alignments
Criteria: 70%, 100 bp
Regions: 339
Alignment 4
P.uncia
Snow leopard
8 alignments
Criteria: 70%, 100 bp
Regions: 328
Alignment 5
P.concolor
Cougar
57 alignments
Criteria: 70%, 100 bp
Regions: 342
Alignment 6
F.nigripes
Black-footed cat
22 alignments
Criteria: 70%, 100 bp
Regions: 230
Alignment 7
P.onca
Jaguar
36 alignments
Criteria: 70%, 100 bp
Regions: 292
Alignment 8
P.yagouaroundi
Yagouaroundi
19 alignments
Criteria: 70%, 100 bp
Regions: 320
Alignment 9
C.caracal
Caracal
25 alignments
Criteria: 70%, 100 bp
Regions: 351
100%
50%
100k
110k
120k
130k
140k
150k

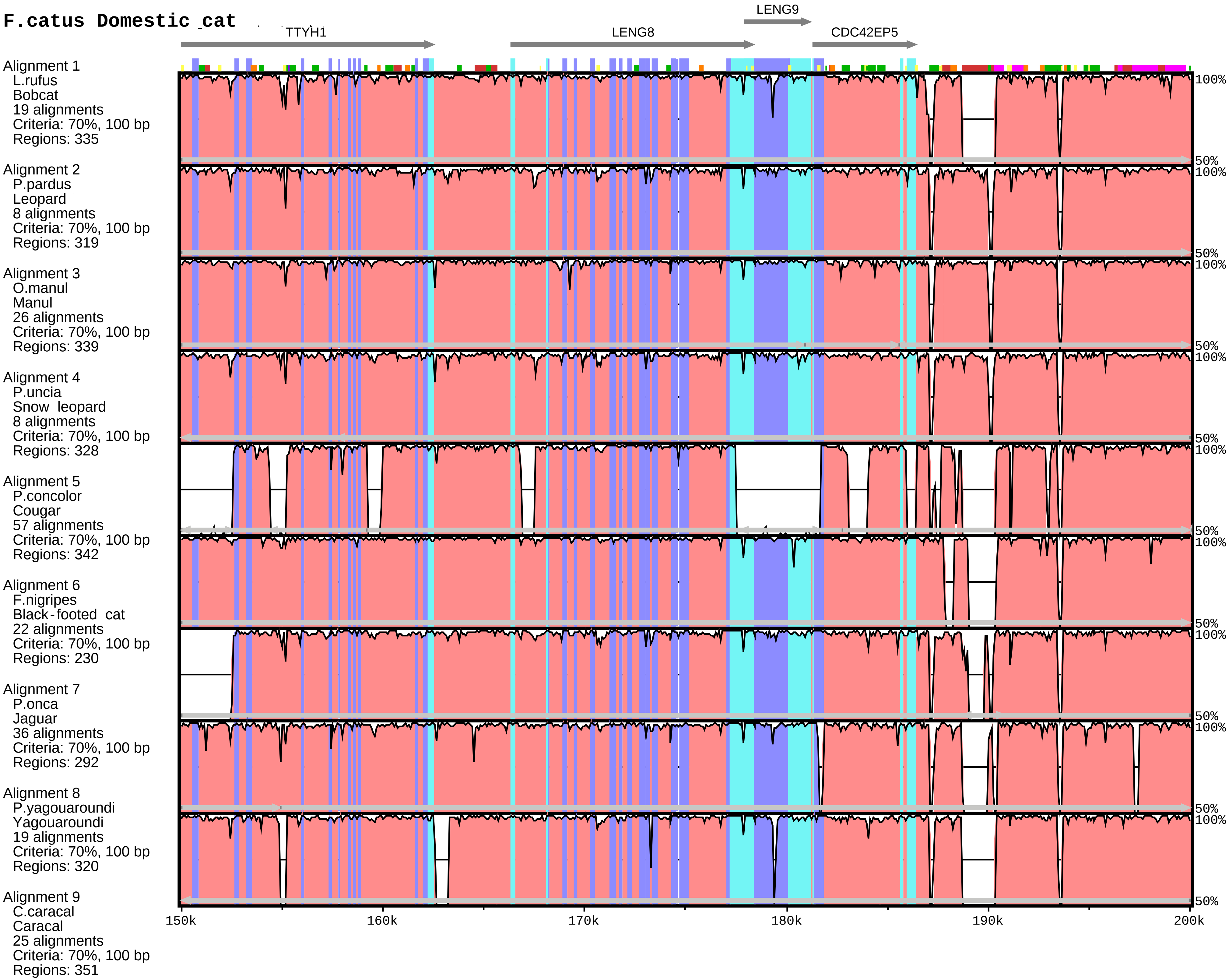
F.catus Domestic cat
TTYH1
LENG8
LENG9
CDC42EP5
Alignment 1
L.rufus
Bobcat
19 alignments
Criteria: 70%, 100 bp
Regions: 335
Alignment 2
P.pardus
Leopard
8 alignments
Criteria: 70%, 100 bp
Regions: 319
Alignment 3
O.manul
Manul
26 alignments
Criteria: 70%, 100 bp
Regions: 339
Alignment 4
P.uncia
Snow leopard
8 alignments
Criteria: 70%, 100 bp
Regions: 328
Alignment 5
P.concolor
Cougar
57 alignments
Criteria: 70%, 100 bp
Regions: 342
Alignment 6
F.nigripes
Black-footed cat
22 alignments
Criteria: 70%, 100 bp
Regions: 230
Alignment 7
P.onca
Jaguar
36 alignments
Criteria: 70%, 100 bp
Regions: 292
Alignment 8
P.yagouaroundi
Yagouaroundi
19 alignments
Criteria: 70%, 100 bp
Regions: 320
Alignment 9
C.caracal
Caracal
25 alignments
Criteria: 70%, 100 bp
Regions: 351
100%
50%
150k
160k
170k
180k
190k
200k

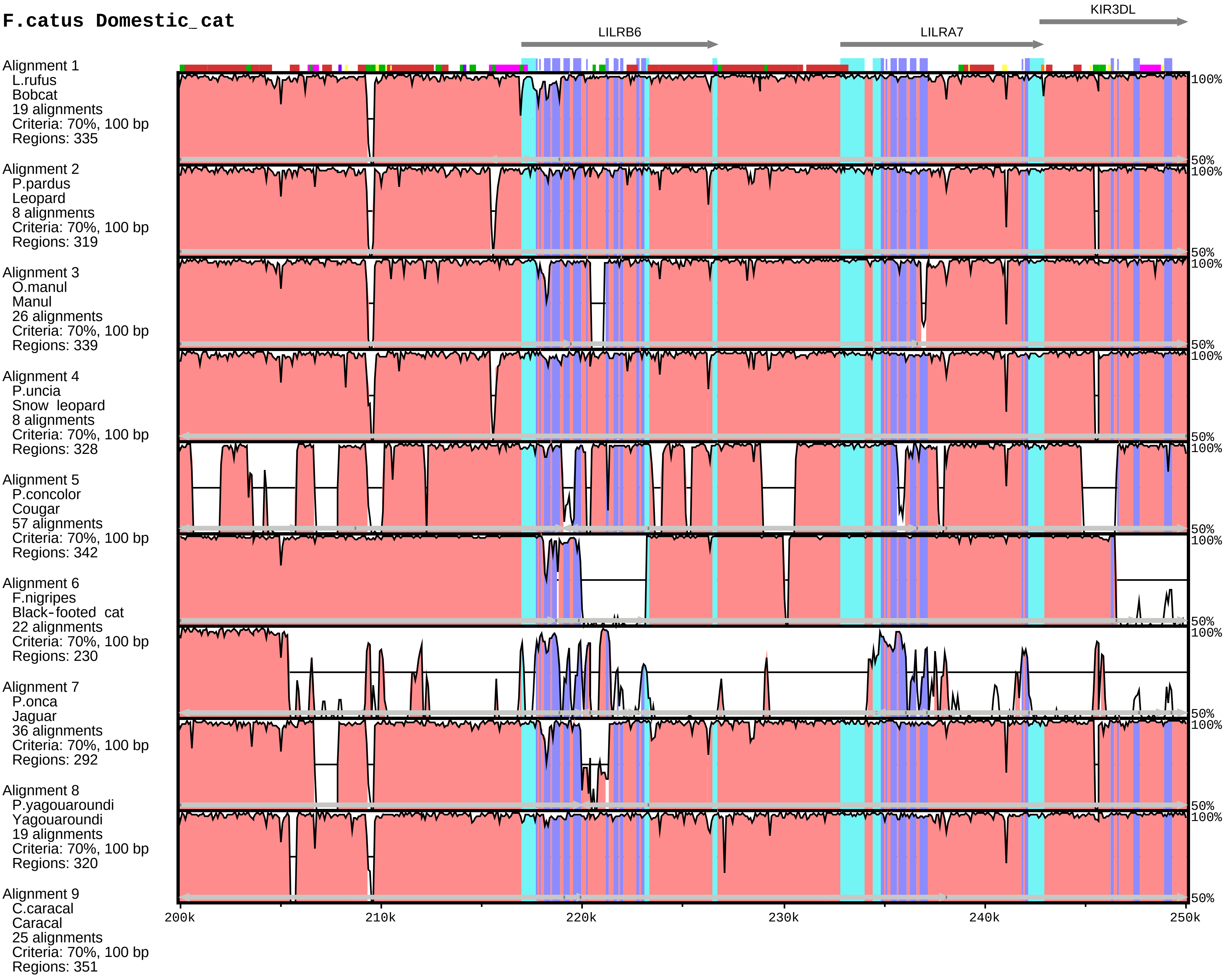
F.catus Domestic_cat
LILRB6
LILRA7
KIR3DL
Alignment 1
L.rufus
Bobcat
19 alignments
Criteria: 70%, 100 bp
Regions: 335
Alignment 2
P.pardus
Leopard
8 alignments
Criteria: 70%, 100 bp
Regions: 319
Alignment 3
O.manul
Manul
26 alignments
Criteria: 70%, 100 bp
Regions: 339
Alignment 4
P.uncia
Snow leopard
8 alignments
Criteria: 70%, 100 bp
Regions: 328
Alignment 5
P.concolor
Cougar
57 alignments
Criteria: 70%, 100 bp
Regions: 342
Alignment 6
F.nigripes
Black-footed cat
22 alignments
Criteria: 70%, 100 bp
Regions: 230
Alignment 7
P.onca
Jaguar
36 alignments
Criteria: 70%, 100 bp
Regions: 292
Alignment 8
P.yagouaroundi
Yagouaroundi
19 alignments
Criteria: 70%, 100 bp
Regions: 320
Alignment 9
C.caracal
Caracal
25 alignments
Criteria: 70%, 100 bp
Regions: 351
100%
50%
200k
210k
220k
230k
240k
250k

F.catus Domestic_cat

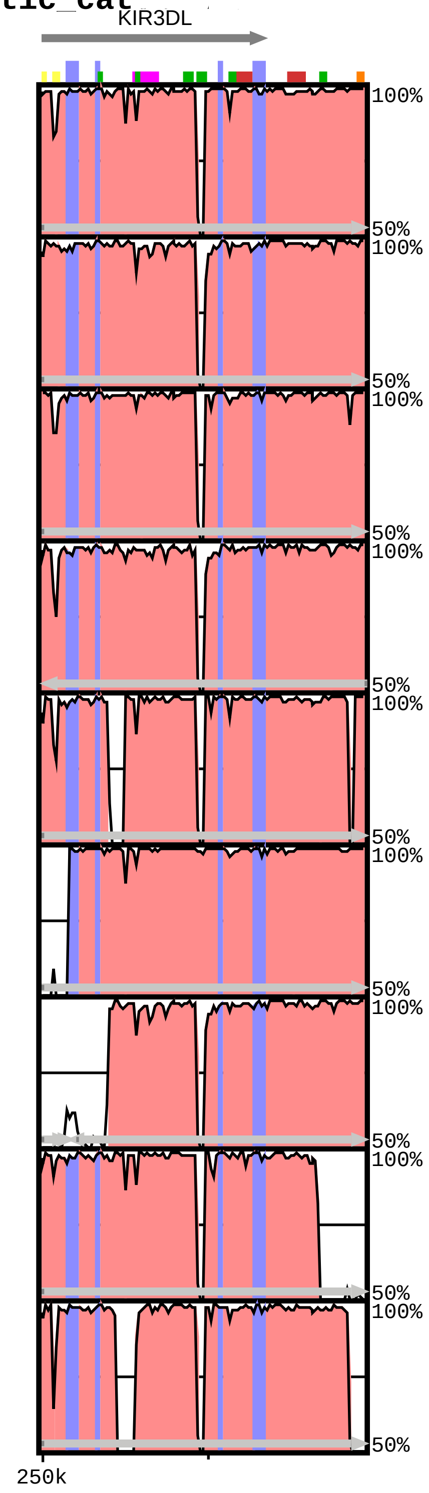

Alignment 1
L.rufus
Bobcat
19 alignments
Criteria: 70%, 100 bp
Regions: 335

Alignment 2
P.pardus
Leopard
8 alignments
Criteria: 70%, 100 bp
Regions: 319

Alignment 3
O.manul
Manul
26 alignments
Criteria: 70%, 100 bp
Regions: 339

Alignment 4
P.uncia
Snow leopard
8 alignments
Criteria: 70%, 100 bp
Regions: 328

Alignment 5
P.concolor
Cougar
57 alignments
Criteria: 70%, 100 bp
Regions: 342

Alignment 6
F.nigripes
Black-footed cat
22 alignments
Criteria: 70%, 100 bp
Regions: 230

Alignment 7
P.onca
Jaguar
36 alignments
Criteria: 70%, 100 bp
Regions: 292

Alignment 8
P.yagouaroundi
Yagouaroundi
19 alignments
Criteria: 70%, 100 bp
Regions: 320

Alignment 9
C.caracal
Caracal
25 alignments
Criteria: 70%, 100 bp
Regions: 351
